# Monitoring intracellular replication dynamics unveils high proportion of non-replicating antibiotic-tolerant *Staphylococcus aureus* inside osteoblasts

**DOI:** 10.1371/journal.ppat.1013525

**Published:** 2025-09-23

**Authors:** Florian C. Marro, Jacques Brocard, Allison Faure, Angélique Sion, Paul O. Verhoeven, Laurie Canonne-Desbiolles, Laurence Conraux, Laura Jaffrelot, Chantal Monlong, Ariel J. Blocker, Nelly Dubarry, Frédéric Laurent, Jérôme Josse

**Affiliations:** 1 Evotec ID Lyon, In Vitro Biology, Infectious Diseases and Antibacterials Unit, Lyon, France; 2 Centre International de Recherche en Infectiologie (CIRI), Inserm U1111, Université Claude Bernard Lyon 1, CNRS UMR5308, ENS Lyon, Lyon, France; 3 PLATIM, Université Claude Bernard Lyon 1, CNRS UAR3444, INSERM US8, ENS Lyon, SFR Biosciences, Lyon, France; 4 Centre National de Reference des Staphylocoques, Institut des Agents Infectieux, Hospices Civils de Lyon, Lyon, France; University of Tubingen, GERMANY

## Abstract

Therapeutic failures and relapses are critical challenges in *Staphylococcus aureus* bone and joint infections. These issues may stem, in part, from the incomplete eradication of *S. aureus* residing within osteoblasts, the bone-forming cells, despite recommended antibiotic treatment. However, the mechanisms underlying intraosteoblastic *S. aureus* survival remain poorly understood. Here, we used automated real-time fluorescence microscopy at the single-host-cell level to monitor the intracellular replication dynamics of clinical *S. aureus* strains and their survivors of rifampicin treatment in MG-63 osteoblast cell line. *S. aureus* replication dynamics was heterogeneous both within and across strains, while survival to rifampicin treatment was uniformly characterized by a non-replicative phenotype. Surprisingly, rifampicin killed less than 0.3 log of intraosteoblastic *S. aureus*, and only during the early phase of infection. The majority of *S. aureus* that survived rifampicin treatment remained non-replicative intracellularly after rifampicin withdrawal, yet they retained the capacity to regrow on agar following release from host cells. This high proportion of non-replicative antibiotic-tolerant *S. aureus* inside osteoblasts may contribute to the high rates of therapeutic failures in bone and joint infections.

## Authors summary

*Staphylococcus aureus* is a major cause of human infections, including bone and joint infections. Therapeutic failures are frequently observed in staphylococcal infections, even in the absence of antibiotic resistance. Traditionally considered an extracellular pathogen, *S. aureus* is capable of being internalized and surviving within host cells, including osteoblasts, the cells that build bones. In this study, we showed *in vitro* that intraosteoblastic staphylococci had heterogeneous intracellular replication dynamics with non-replicating bacteria and some that started to replicate after variable delays. We demonstrated that rifampicin killing is limited but prevented *S. aureus* outgrowth with survivors remaining in a non-replicative profile. These findings suggest the need for novel therapeutic strategies to target the intracellular, tolerant non-replicative *S. aureus* reservoir that may drive treatment failures in staphylococcal infections.

## Introduction

*Staphylococcus aureus* is a Gram-positive commensal bacterium known as an opportunistic pathogen causing both human nosocomial and community-acquired infections. Disease severity ranges from local and superficial skin and soft tissue infections to serious invasive infections, including sepsis, pneumonia, endocarditis, and bone and joint infections (BJIs) [1–4]. BJIs are polymorphic and difficult-to-treat infections notably including native joint septic arthritis, chronic osteomyelitis and orthopedic device-related infections [5]. *S. aureus* is the most incriminated pathogen in BJIs. Despite intensive therapy combining antibiotic treatment and surgical management, the rate of staphylococcal BJIs therapy failure reaches up to 24% [6–8]. Biofilm formation, phenotypic switch to small colony variant and intracellular survival have been suggested as major *S. aureus* mechanisms in the chronicization and relapse of BJIs [5,9].

Originally considered as an exclusive extracellular pathogen, evidence has demonstrated that *S. aureus* can adhere, invade, and survive in both professional phagocytes and non-professional phagocytic host cells [10]. In the context of BJIs, *S. aureus* invasion of osteoblasts (bone-forming cells), osteocytes (bone matrix-embedded cells), and osteoclasts (bone-resorbing cells) has been observed *in vitro* but also in clinical samples for osteoblasts and osteocytes and *in vivo* for osteoclasts [11–16]. A recent study, using fixed microscopy, demonstrated that the intraosteoblastic replication of *S. aureus* is strain-dependent [17]. Some strains are non-replicative while other strains demonstrate intense replication leading to lysis of the host cell. However, only a very limited number of studies have monitored the replication dynamics of intracellular *S. aureus* in real-time, and none analyzed it quantitatively at the single-cell level [13,18–24].

Numerous studies have investigated the efficacy of antibiotics to eliminate intracellular *S. aureus* inside osteoblasts and other cell types [25,26]. These studies showed, consistently with clinical data, that rifamycins and fluoroquinolones are among the best antibiotics to eliminate intracellular *S. aureus* [26–32]. However, in any case, intracellular *S. aureus* was never completely eradicated with antibiotics at clinical concentrations. Many factors could contribute to the incomplete clearance of intracellular *S. aureus* by antibiotics. These include poor antibiotic penetration into the host cells and the presence of antibiotic-tolerant bacteria exhibiting a persisters phenotype characterized by a transient non-growing state conferring antibiotic tolerance without the need for a genetic modification [33,34]. A study has shown the presence of intracellular *S. aureus* persisters following oxacillin, clarithromycin, and moxifloxacin treatments using end-point flow cytometry [35]. However, the relationship between the replication dynamics of intraosteoblastic *S. aureus* and the tolerance to BJI-relevant antibiotics has yet to be fully elucidated.

Here, using automated real-time fluorescence microscopy, we observed that the intracellular replication dynamics of *S. aureus* in MG-63 osteoblast cell line was heterogeneous, varying both between host cell and across bacterial strain. We found that a significant proportion of infected osteoblasts contained *S. aureus* that remained non-replicative throughout the duration of infection. This proportion can concern almost 85% of infected osteoblasts depending on the strain studied. Additionally, we highlighted different intracellular replication patterns and intensities reinforcing a specific strain-dependent profile. Then, intraosteoblastic *S. aureus* were challenged with rifampicin and ciprofloxacin alone or in combination at clinical concentration. We demonstrated that rifampicin had a limited impact and that survivors were all strain independently non-replicating. Additionally, we stressed out that ciprofloxacin treatment altered the intracellular replication dynamics in a dose-dependent manner.

## Results

### Monitoring intracellular replication dynamics revealed a high proportion of non-replicative *Staphylococcus aureus* inside osteoblasts

**Fig 1:**
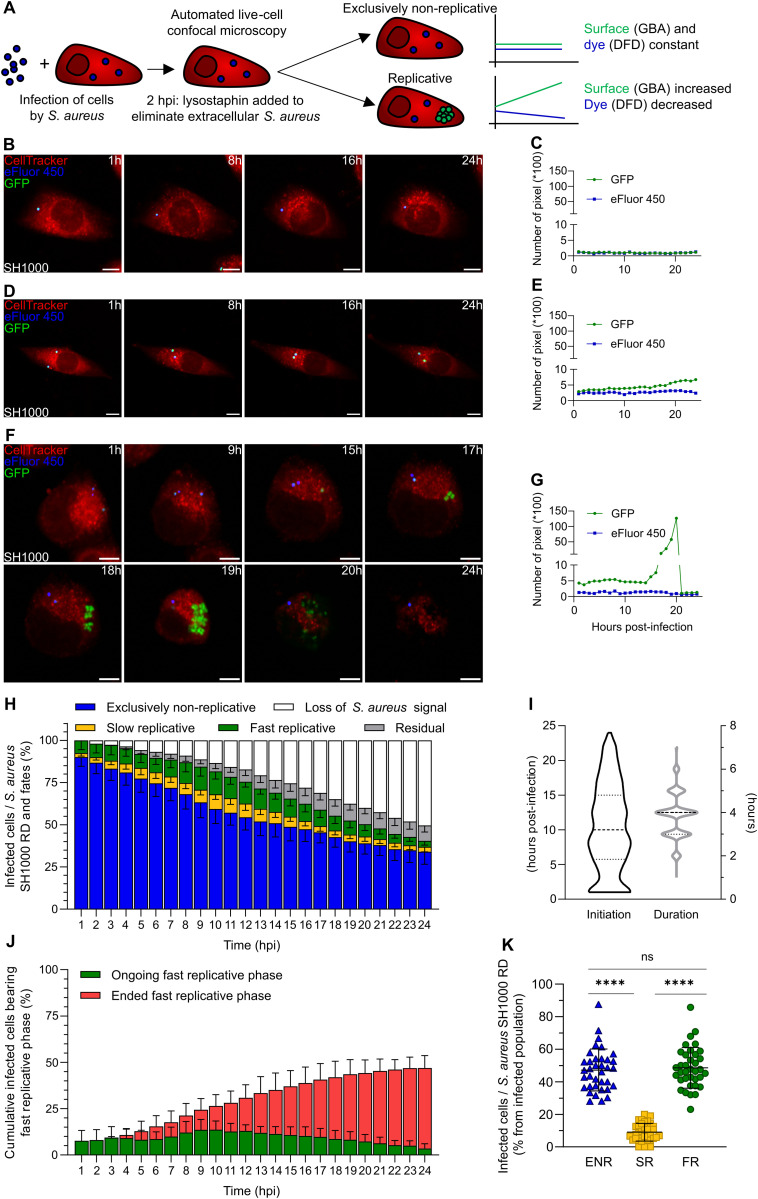
Intracellular *Staphylococcus aureus* replication dynamics is heterogeneous over time. **(A)** Experimental design for assessing *S. aureus* replication dynamics in MG63 osteoblastic cells. **(B-K)** MG63 cells, seeded at sparse density and labeled with CellTracker Red CMTPX (red), were infected at MOI 8 with *S. aureus* SH1000 expressing GFP (green) pre-labeled by eFluor-450 (blue). Following 2 hours of co-incubation, lysostaphin at 10 µg/mL was added to eliminate extracellular *S. aureus*. Time-lapse imaging was conducted over 24 hours with hourly acquisitions using automated confocal microscopy. **(B-G)** Representative confocal images and corresponding quantification of green (GFP) and blue (eFluor-450) fluorescence intensities over time. Images show single osteoblastic cells infected by: exclusively non-replicative *S. aureus* **(B, C**), or at least one *S. aureus* transitioning from quiescence to slow replication **(D, E)** or fast replication leading to host cell lysis **(F, G)** (scale bar = 10 µm). **(H)** Hourly quantification of infected cells based on intracellular *S. aureus* replication dynamics (RD) across the 24-hour infection period. **(I)** Initiation and duration of the fast replicative phases. **(J)** Hourly quantification of infected cells experiencing either an ongoing or an ended fast replicative phase. **(K)** Global quantification of infected cells based on intracellular *S. aureus* replication dynamics over an infection period of 24 hours (ENR: Exclusively non-replicative; SR: slow replicative; FR: fast replicative). **(H-K)** Results were presented as mean ± SD **(H, J, K)** or median and quartiles **(I)**, representing 36 individual values **(H, J, K)** from 12 independent experiments (**H**, **K**: N = 1005 infected cells, **I**, **J**: N = 474 infected cells). Mann-Whitney test: ****p < 0.0001.

To investigate the correlation between intraosteoblastic replication dynamics of *Staphylococcus aureus* and antibiotic tolerance, we first set up an automated real-time fluorescence microscopy approach. We combined two previously established analysis models: (*i*) the global bacterial area [19,36,37] (GBA) method based on the principle that the surface area of a bacterial population is related to the number of individuals in that population and (*ii*) the dye fluorescence dilution [20,38] (DFD) method relying on a two-fold dilution of the individual initial bacterial content at each replicative step (Fig 1A). The GBA method was employed to monitor the replication dynamics while the DFD method allowed us to highlight non-replication. When intracellular replication occurs, the total GFP surface area relying on the number of individuals increases (GBA), while the eFluor-450 intensity decreases (DFD). In contrast, if there is no intracellular replication, the GFP surface area (GBA) and the eFluor-450 intensity (DFD) will remain constant (Fig 1A). For these implementation experiments, we used the *S. aureus* GFP-expressing SH1000 reference strain that we labelled with eFluor-450 prior to the experiment. Homogeneous and sustained expression of constitutive GFP as well as the effectiveness of eFluor-450 were validated by flow cytometry prior to microscopy experiments (S1 Fig). We also ensured that the presence of the plasmid and the pre-infection eFluor-450 labeling did not impact the internalization and intracellular survival of *S. aureus* SH1000 inside osteoblasts (S2 Fig).

Following 24-hour monitoring with image acquisition every hour, intracellularly infected osteoblasts were categorized into three subsets according to the *S. aureus* replication dynamics: i) osteoblasts containing exclusively non-replicative *S. aureus* associated with a stable or decreasing level of GFP signal as long as the cell is infected, (Fig 1B, 1C and S1 Movie), ii) osteoblasts containing at least one *S. aureus* entering a slow replication phase associated with a slight constant increase in GFP signal, (Fig 1D, 1E and S2 Movie), iii) osteoblasts containing at least one *S. aureus* entering a fast replication phase associated with a brief surge in GFP signal, (Fig 1F, 1G and S3 Movie). Time-lapse monitoring confirmed stable constitutive GFP signal of intracellular non-replicative *S. aureus* (Fig 1C and 1G). Most of the infected cells contained exclusively non-replicative *S. aureus* at the beginning of the monitoring (Fig 1H; average, 90.07% ± 5.47). Intraosteoblastic fast replicative events could be detected as early as 1-hour post-infection (hpi) (Fig 1I and 1J). Replication, either slow or fast, was observed throughout the time course of the infection (Fig 1H-1J). It led to a decreased proportion of osteoblasts harboring exclusively non-replicative *S. aureus*. Most of the fast replicative phases lasted between 3 hours and 5 hours and all initiated fast replicative phases led to the host cell lysis (Fig 1F, 1G, 1I and 1J). Consequently, we observed a gradual decrease of infected cell numbers, reaching 49.19% ± 8.11 of the initial infected osteoblast population at 24 hpi (Fig 1H). Interestingly, some *S. aureus* could still reside within the host cell aggregates after its lysis, sometimes until the end of the acquisition (Fig 1F). We termed this case “residual” (Fig 1H). The loss of *S. aureus* signal can be mostly attributed to the host cell lysis after replication. However, we cannot exclude a complete clearance of intracellular non-replicative *S. aureus* by osteoblasts as observed in rare cases. At 24 hpi, almost half of the infected osteoblasts still contained exclusively non-replicative *S. aureus* (Fig 1H and 1K; average 47.7% ± 7.75) whereas the other half had contained *S. aureus* experiencing a fast replicative phase (Fig 1J and 1K; average 47.08% ± 9.3). Few infected osteoblasts were associated with slow replication of *S. aureus* (Fig 1K; average 5.66% ± 5.09). Moreover, we observed that more than half of the osteoblasts containing fast replicative *S. aureus* concurrently carried non-replicative *S. aureus* that maintained eFluor-450 fluorescence intensity levels (Fig 1F and 1G; average 63.71% ± 11.03). Therefore, almost 78% of the infected cells served as niches for non-replicative *S. aureus*, either exclusively or in a shared manner with replicating bacteria. Interestingly, the use of lower MOIs did not impact on the *S. aureus* replication dynamics and intracellular bacterial load at 1 hpi while it significantly decreased the percentage of infected cells (S3 Fig).

To determine whether this replication pattern is strain specific, we assessed the intracellular replication dynamics of different *S. aureus* strains (S1 Table). We included the reference strain HG001, which is routinely used in the laboratory to test the antibiotic’s impact [27,39] and an isogenic mutant of *S. aureus* SH1000 deleted for *agrA*, as a non-replicative reference [17]. It was previously shown that the deletion of *agrA*, a gene of the virulence regulator agr operon involved in a major quorum sensing system known to impact the *S. aureus* vacuolar escape, resulted in limited intracellular replication [10,17]. The collection also included a selection of 6 *S. aureus* clinical isolates collected from patients experiencing BJIs [27]. These isolates were previously phenotypically characterized using end-point fixed microscopy and are representative of three distinct replication and cytotoxic profiles (S1 Table) [17]. All these strains were able to invade and survive inside osteoblasts (S4 Fig).

**Fig 2:**
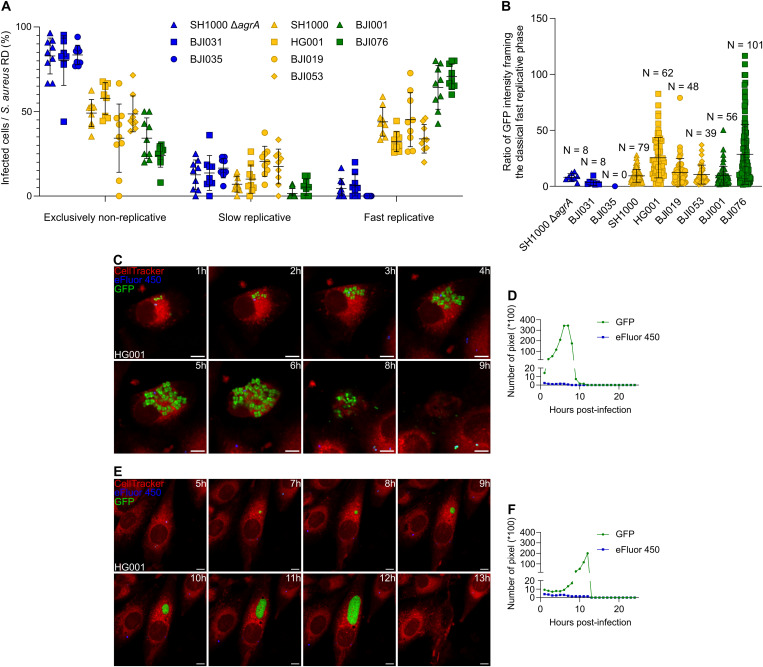
Intracellular *Staphylococcus aureus* replication dynamics is heterogeneous among clinical isolates. **(A-F)** MG63 cells (red) were seeded, labeled and infected as previously described in Fig. 1 with a range of *S. aureus* strains and clinical isolates expressing GFP (green) and pre-labeled by eFluor-450 (blue), (S1 Table). Time-lapse imaging was conducted over 24 hours with hourly acquisitions using automated confocal microscopy. **(A)** Quantification of infected cells based on intracellular *S. aureus* replication dynamics (RD) over an infection period of 24 hours. (Classification: profile mainly non-replicative (blue); intermediate replicative profile (yellow); mainly replicative profile (green)). **(B)** Quantification of the classical fast replicative phase intensities, that represent the increase in *S. aureus* population size, through the ratio of total green (GFP) pixel count from replication onset to host cell lysis. **(C-F)** Representative confocal images and corresponding quantification of green (GFP) and blue (eFluor-450) fluorescence intensities over time. Images show single osteoblastic cells infected by *S. aureus* experiencing a classical fast replicative phase either in an irregular shape (**C**, **D**: strain HG001), or constrained shape (**D**, **E**: strain HG001), (scale bar = 10 µm). **(A, B)** Results were presented as mean ± SD representing 9 individual values **(A)** from 3 independent experiments (**A**: N = 1631 infected cells, **B**).

Distinct intracellular replication profiles emerged among this collection of *S. aureus* strains that we classified into 3 groups (Figs 2A and S5). Strains ranked as group I had a mainly non-replicative profile with more than 80% of infected cells harboring exclusively non-replicative *S. aureus* (Figs 2A and S5; strains in blue: SH1000 *Δ*agrA**, BJI031, and BJI035). As expected, deletion of *agrA* in the SH1000 strain significantly favored a non-replicative phenotype when compared to its wild-type counterpart. Four strains, including the two reference strains, SH1000 and HG001, clustered into group II having an intermediate replicative profile with less than half of the infected osteoblasts containing fast replicative bacteria (strains in yellow: SH1000, HG001, BJI019, and BJI053). Finally, group III had a high replication profile with more than 64% of infected osteoblasts containing fast replicative bacteria (strains in green: BJI076 and BJI001). Corroborating these findings, no significant cytotoxicity was observed for the group I strains, while group II and III strains induced significant cytotoxicity (S6 Fig).

Through this multi-strain analysis, we observed that *S. aureus* HG001 (group II) and BJI076 (group III) experienced more replication steps before host cell lysis compared to the other strains (Fig 2B). It matched with a longer duration of fast replicative phases as compared to the other strains (S7 Fig). Interestingly, fast replicative phases displayed heterogeneous shapes, ranging from irregular to constrained circular forms, even within the same strain (Fig 2C-2F; S4 and S5 Movies).

**Fig 3:**
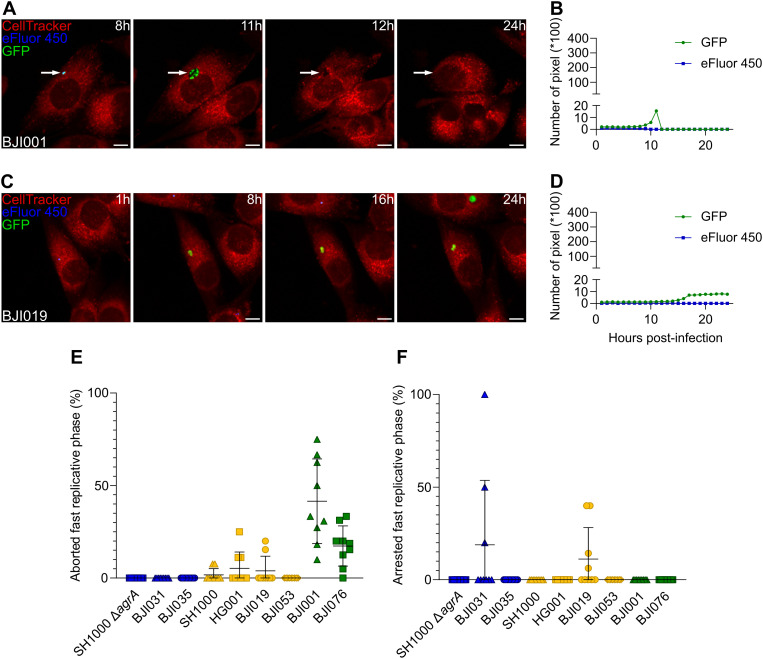
Fast replicative phases are heterogeneous. **(A-F)** MG63 cells (red) were seeded, labeled and infected as previously described in Fig 1 with a range of *S. aureus* strains and clinical isolates expressing GFP (green) and pre-labeled by eFluor-450 (blue), (S1 Table). Time-lapse imaging was conducted over 24 hours with hourly acquisitions using automated confocal microscopy. **(A-D)** Representative confocal images and corresponding quantification of green (GFP) and blue (eFluor-450) fluorescence intensities over time. Images show single osteoblastic cells infected by *S. aureus* experiencing either: an aborted fast replicative phase (**A**, **B**: strain BJI001), or an arrested fast replicative phase (**C**, **D**: BJI019) (scale bar = 10 µm). **(E**, **F)** Quantification of the proportion of aborted **(E)** and arrested **(F)** fast replicative phases relative to the total fast replicative events measured. **(E**, **F)** Results were presented as mean ± SD representing 9 individual values from 3 independent experiments.

We also observed two alternative fast replicative patterns with strain-dependent occurrence. We termed them “aborted” and “arrested” which we differentiate from the “classical” fast replication pattern that we described above. The aborted fast replicative pattern was characterized by a rapid replication phase followed by the sudden disappearance of the GFP bacterial signal within a single time-point interval, without immediate lysis of the host cell (Fig 3A and 3B; S6 Movie). This pattern was observed for 5 strains out of 9, and accounted for up to 41.5% of the fast replicative phases for *S. aureus* BJI001 (Fig 3E). The arrested fast replicative pattern involved an initial fast replicative phase followed by a complete growth arrest (Fig 3C and 3D; S7 Movie). This pattern was observed only in strains BJI019 (group II) and BJI031 (group I), representing up to 18.8% of the total fast replicative phases (Fig 3F).

As a common feature all the strains from our panel performing fast replicative phases displayed a gradual transitioning from quiescence to replication (S7 Fig). Moreover, for all the tested strains regardless of their group, osteoblasts carrying exclusively non-replicative *S. aureus* consistently represented the predominant population at each time point of the kinetics (S5 Fig). This occurs despite some strains exhibiting an overall predominance of osteoblast carrying replicative *S. aureus* and reflects the transient nature of the fast replicative phase, which ends with host cell lysis and release of intracellular *S. aureus*.

We observed that most strains showed a decrease in CFU at 24 hpi compared to 1 hpi (S4 Fig). For group I strains, the small yet significant reduction likely reflects the survival of non-replicative *S. aureus* in viable host cells, with limited clearance by osteoblast. In groups II and III, the more pronounced CFU decrease is consistent with classical fast replicative phases leading to host cell lysis and bacterial release into the extracellular killing environment. Interestingly, strain BJI019 stood out by showing an increase in CFU at 24 hpi (S4 Fig). This is consistent with its replicative dynamics, BJI019 exhibited the highest number of ongoing fast replicative phases at 24 hpi resulting in a large bacterial load in intact host cells at the time of collection (S5 Fig). Additionally, this strain displayed a subset of arrested fast replicative phases (Fig 3F).

Overall, these data emphasize replication dynamics heterogeneity with a proportion of infected osteoblasts containing exclusively non-replicative *S. aureus* that can reach more than 80% depending on the strain studied.

### Intraosteoblastic rifampicin-surviving *Staphylococcus aureus* display a non-replicative, strain-independent profile

We then investigated if the absence of replication can be correlated with antibiotic tolerance by challenging intraosteoblastic *S. aureus* with rifampicin. For these experiments, we used the clinical bone concentration of rifampicin (6 µg/mL), corresponding to approximately 1,000xMIC. At this concentration, rifampicin is rapidly bactericidal against planktonic bacteria in the exponential phase whereas stationary phase bacteria survived rifampicin up to 10,000xMIC concentrations (S8 Fig). Moreover, to ensure that a bactericidal concentration is achieved at the intraosteoblastic level, penetration and retention of rifampicin were measured over a 24-hour treatment period. Intracellular concentration reached almost 1.5 µg/mL at 8 hours before decreasing but was still over the MIC at 24 hours (S9A Fig).

**Fig 4:**
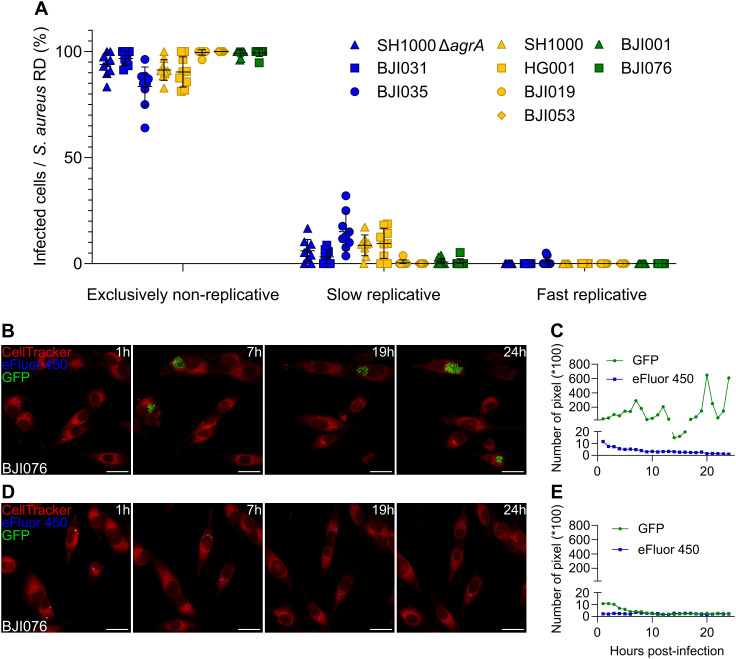
Intracellular *Staphylococcus aureus* survivors to rifampicin are non-replicative, regardless of strain. **(A**-**E)** MG63 cells (red) were seeded, labeled and infected as previously described in Fig 1 with a range of *S. aureus* strains and clinical isolates expressing GFP (green) and pre-labeled by eFluor-450 (blue), (S1 Table, BJI035: rifampicin resistant). Following 2 hours of co-incubation, lysostaphin at 10 µg/mL was added to eliminate extracellular *S. aureus*. Concomitantly, cells were treated with rifampicin at 6 µg/mL. Time-lapse imaging was conducted over 24 hours with hourly acquisitions using automated confocal microscopy. **(A)** Quantification of infected cells based on intracellular *S. aureus* replication dynamics (RD) over an infection period of 24 hours. **(B-E)** Representative confocal images and corresponding quantification of green (GFP) and blue (eFluor-450) fluorescence intensities of control (**B**, **C**) and rifampicin-treated conditions (**D**, **E**) over time. Images show population of osteoblastic cells infected by *S. aureus* BJI076 experiencing either quiescent and replicative states **(B)** or only quiescence **(D)**, (scale bar = 40 µm). **(A)** Results were presented as mean ± SD representing 9 individual values from 3 independent experiments (N = 1848 infected cells).

Under rifampicin treatment, the percentage of infected osteoblasts bearing exclusively non-replicative *S. aureus* was predominant throughout the kinetics and reached 90.5% to 100%, depending on the strain (Figs 4A–4E and S10). In contrast, the fast replicative phases were no longer detected while osteoblasts carrying slow replicative *S. aureus* ranged from 0% to 9.5% (Figs 4A–4E; S8 and S9 Movies). The slow replicative phases observed were not sustained and the bacterial signal rapidly disappeared due to killing by rifampicin (S10 Fig). Only the replicative pattern of the BJI035 strain stood out from the others, which is likely due to its rifampicin resistance profile.

**Fig 5:**
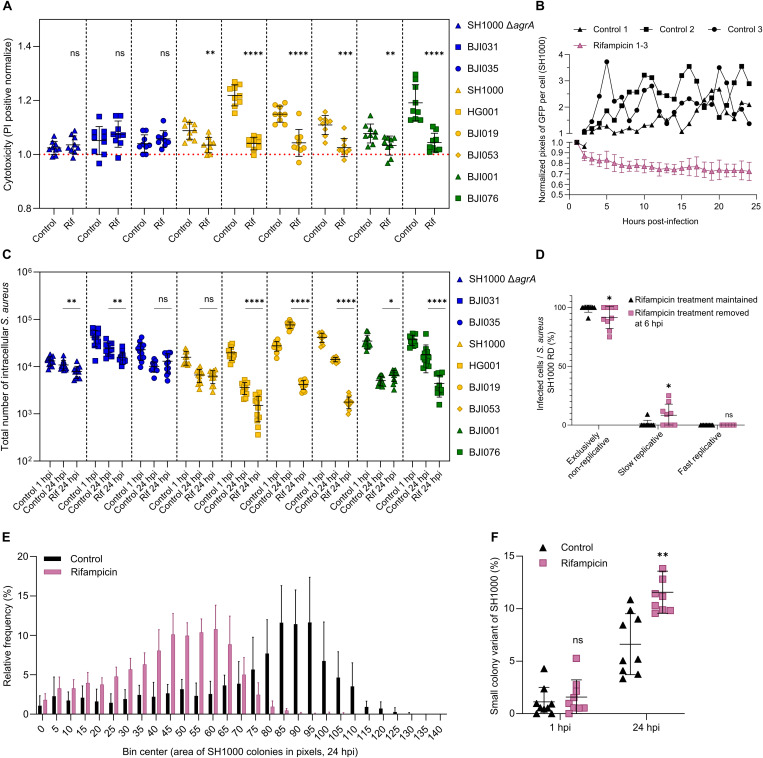
Rifampicin treatment rescue host cells from lysis and *S. aureus* survivors regrow on agar plate with a global reduce colony size. MG63 cells labeled **(B**, **D)** or not (**A**, **C**, **E**) with CellTracker Red CMTPX (red) were seeded at sparse **(B**, **D)** or confluent **(A**, **C**, **E)** density and infected and treated as previously described in Fig 4 (BJI035: rifampicin resistant). **(A)** At 2 hpi propidium iodide (PI) was added at 2 µg/mL. At 24 hpi PI fluorescence intensity was measured with a plate reader. PI fluorescence intensity is normalized to uninfected cells in untreated and rifampicin-treated conditions. **(B)** Time-lapse imaging was conducted over 24 hours with hourly acquisitions using automated confocal microscopy. Quantification of the *S. aureus* SH1000 population size per cell over time represented by the total green (GFP) pixel count normalized per cell. **(C, E)** Intracellular *S. aureus* SH1000 were collected at 1 hpi and 24 hpi, and the total number of *S. aureus* forming colonies on agar plate was investigated **(C)** as well as their area distribution frequency at 24 hpi **(E)** and the small colony variants rates **(F)**. **(D)** At 6 hpi, rifampicin treatment was either withdrawn by washing or maintained, and incubation continued. Time-lapse imaging was conducted over 18 hours post-withdrawal with hourly acquisitions using automated confocal microscopy. Quantification of infected cells based on intracellular *S. aureus* replication dynamics (RD). Results were presented either as single value and mean ± SD from 3 independent experiments **(B, E)** or as mean ± SD representing 9 individual values from 3 independent experiments **(A**, **D**: N = 165 infected cells, **F)** or 12 individual values from 4 independent experiments **(C)**. One-way ANOVA with Dunnett’s correction for multiple post hoc comparisons with the control **(A)** and Mann-Whitney test **(C**, **D, F)**: *p < 0.05, **p < 0.01, ****p < 0.0001.

Corroborating these findings, rifampicin treatment, by erasing the intraosteoblastic *S. aureus* fast replicative phase, rescued the cells from lysis as shown by propidium iodide incorporation cytotoxicity monitoring (Fig 5A).

Follow-up of the intracellular GFP signal in the control condition exhibited an up and down profile reflecting the fast replicative phase patterns observed for the group II and III (Figs 5B and S11). The curves did not match between independent experiments, indicating that the kinetics of *S. aureus* entering fast replication was experiment-dependent. Conversely, the GFP signal per cell decreased in rifampicin-treated infected cells supporting the absence of fast replication and killing. Interestingly, rifampicin-mediated killing never exceeded a 0.3 log reduction and was observed exclusively during the first 10 hours of the kinetics for all sensitive clinical strains (S12 Fig). It suggests that rifampicin induces a dormant-like state that locks bacteria in a non-replicative, self-tolerant condition, preventing later replication observed in controls.

To complete our observations, we investigated if rifampicin withdrawal during the intracellular infection can revert the non-replicative phenotype. To this end, infected osteoblasts by *S. aureus* SH1000 and exposed to rifampicin were washed at 6 hpi to remove the antibiotic treatment. The efficacy of the washing step was assessed by mass spectrometry (S9A Fig). Upon rifampicin treatment withdrawal, a discrete yet significant increase in the percentage of osteoblasts harboring a slow replicative phase was observed, from 1.01% ± 3.03 to 8.42% ± 9.39 thus decreasing to the same extent the subset of osteoblasts containing exclusively non-replicative *S. aureus* (Fig 5D). However, most intraosteoblastic *S. aureus* remained non-replicative despite withdrawal of rifampicin and no fast replicative phase was observed.

CFU experiments confirmed that 24-hour rifampicin treatment failed to clear host cells from each strain and that survivors could regrow on agar plates (Fig 5C). Interestingly, intracellular rifampicin efficacy did not entirely correlate with replication dynamics observed by live imaging. This discrepancy likely stems from replicative temporal differences in the untreated control condition (S5 and S11 Figs). For example, within group III, BJI001 had already passed its replication peak at the time of intracellular *S. aureus* collection, while BJI076 was still undergoing active replication phase, leading to higher CFU counts in the control and thus an increased apparent efficacy of rifampicin compared to BJI001.

Then, the size of the colonies was investigated to highlight the presence of small colony variants (SCV). This phenotype (colonies with a reduced size on plates) is known to be associated with chronic infection and intracellular survival [40–42]. A 24-hour intracellular stay significantly increased the percentage of SCVs and colony area heterogeneity of *S. aureus* SH1000 from both non-treated and treated infected osteoblasts (Figs 5F and S13). Moreover, at 24 hpi, the SCVs rate was significantly higher, and the colony population size was reduced after an intracellular stay with rifampicin treatment compared to the non-treated condition (Fig 5E and 5F).

Electron microscopy experiments revealed that isolated or small clusters of 2–3 intraosteoblastic *S. aureus* were found mainly within vacuoles at 24 hpi with or without rifampicin treatment (S14A–S14E and S14G–S14I Fig) while few localized in the cytosol (S14F Fig). Most of these vacuoles were single-membraned, mainly tight around the bacteria (S14C–S14E, S14G, S14H and S14J Fig) while others were loose multi-membrane systems (S14A and S14B Fig).

Overall, we demonstrated that the rifampicin-mediated killing was limited, happened early in the kinetics, and that the survivors were non-replicative. These bacteria remained generally non-replicative intracellularly after withdrawal of rifampicin while they could regrow on agar with an SCV-like phenotype.

### Ciprofloxacin treatment altered the intracellular replication dynamics in a dose-dependent manner

**Fig 6:**
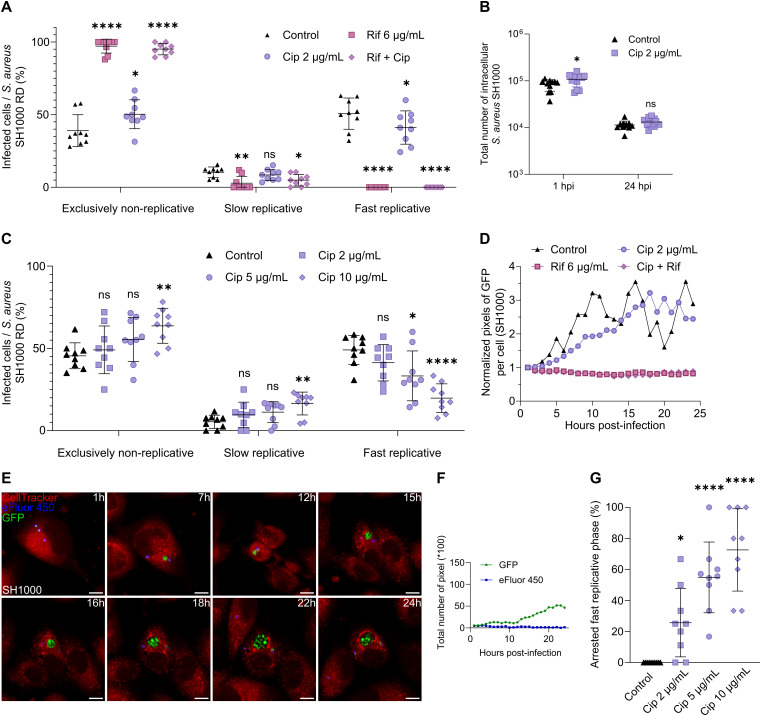
Arrested fast replicative phases are induced by ciprofloxacin treatment. MG63 cells labeled **(A**, **C**-**G)** or not **(B)** with CellTracker Red CMTPX (red) were seeded at sparse density and infected at MOI 8 with *S. aureus* SH1000 expressing GFP (green) pre-labeled by eFluor-450 (blue). Following 2 hours of co-incubation, lysostaphin at 10 µg/mL was added to eliminate extracellular *S. aureus*. Concomitantly, cells were treated with rifampicin at 6 µg/mL and/or ciprofloxacin at 2, 5, or 10 µg/mL or left untreated. Time-lapse imaging was conducted over 24 hours with hourly acquisitions using automated confocal microscopy **(A**, **C**-**G)** or intracellular *S. aureus* were collected at 1 hpi and 24 hpi, and total number of *S. aureus* forming colonies on agar plate was investigated **(B)**. **(A**, **C)** Quantification of infected cells based on intracellular *S. aureus* replication dynamics (RD) over an infection period of 24 hours. **(B)** Total number of intracellular *S. aureus* SH1000 forming colonies on plates. **(D)** Quantification of the *S. aureus* population size per cell over time represented by the total green (GFP) pixel count normalized per cell. Represent 1 experiment of 3 for easier readability. **(E**, **F)** Representative confocal images **(E)** and corresponding quantification of green (GFP) and blue (eFluor-450) fluorescence intensities over time **(F)**. Images show a single osteoblastic cell infected by *S. aureus* SH1000 experiencing an arrested fast replicative phase under ciprofloxacin treatment at 2 µg/mL (scale bar = 10 µm). **(G)** Quantification of the proportion of arrested fast replicative phases relative to the total fast replicative events measured. Results were presented either as mean ± SD representing 9 individual values from 3 independent experiments (**A**: N = 1288, **C**: N = 745, **G**: N = 258 infected cells) or as 12 individual values from 4 independent experiments **(B)**. One-way ANOVA with Dunnett’s correction for multiple post hoc comparisons with the control **(A**, **C**, **G)** or Two-way ANOVA test with Sidak’s correction for multiple comparisons post hoc test (**B**; *p* < 0.05: treatment, *p* < 0.0001: time): *p < 0.05, **p < 0.01, ****p < 0.0001.

To deepen our understanding, we continued our experiments by challenging intraosteoblastic *S. aureus* with ciprofloxacin and a combination of rifampicin and ciprofloxacin which are part of the standard-of-care, notably for prosthetic joint infections [30–32,43]. Combining ciprofloxacin at clinical bone concentration with rifampicin did not impact intraosteoblastic replicative dynamics of *S. aureus*, nor colony size nor SCVs rates, compared to rifampicin alone (Figs 6A and S13). However, ciprofloxacin alone at clinical concentration significantly altered the *S. aureus* replication dynamics when compared to the non-treated condition, slightly increasing the percentage of infected osteoblasts containing non-replicative *S. aureus* as well as slightly decreasing the percentage of infected osteoblasts containing fast replicative *S. aureus* (Fig 6A). This different result from rifampicin can be explained in particular by the poorer penetration of ciprofloxacin, not allowing a concentration above the MIC to be maintained throughout the infection with an extracellular challenge of 2 µg/mL (S9 Fig). CFU experiments revealed no difference between treated and untreated conditions, with no impact on colony size and SCVs rates (Figs 6B and S13). To confirm the concentration dependence of our results, intraosteoblastic *S. aureus* were challenged with ciprofloxacin at an intermediate (5 µg/mL) and clinical plasma concentration (10 µg/mL). It led to an increase in the percentage of osteoblasts bearing exclusively non-replicative *S. aureus* counterbalanced by a decrease of osteoblasts bearing fast replicative *S. aureus*, both in a concentration dependent manner (Fig 6C).

Also, we were intrigued by the pattern of GFP signal under ciprofloxacin treatment, revealing a constant increase of GFP pixels over time with no sudden decrease as observed in the non-treated condition (Fig 6D). Closer observations of replication dynamics showed that some of the fast replicative events were in fact, arrested fast replication phases, as observed previously for some clinical strains (Figs 6E, 6F and 3F). However, in this case, ciprofloxacin induced a significant and dose-dependent proportion of arrested replication phases, representing up to 72.59% ± 26.55 of total fast replicative phases under treatment at plasma concentration (Fig 6G; S10 Movie).

Together, these data emphasize that ciprofloxacin treatment promoted survival of non-replicative *S. aureus* and arrested fast replicative phases in a concentration-dependent manner.

## Discussion

Staphylococcal BJIs are difficult-to-treat infections. Even in the context of appropriate medical and surgical management and optimal antibiotic treatment, the persistence of *S. aureus* in the bone tissue can lead to the chronicization of the infection or to relapsing infections [5]. Antimicrobial resistance cannot explain all the difficulties in treating BJIs. For example, most of the *S. aureus* BJIs are associated with classical methicillin-susceptible strains (MSSA) in France, without specific resistance acquisitions [44,45]. Treatment failures may be associated with specific mechanisms such as internalization and intracellular survival of *S. aureus* inside osteoblasts [12,46].

**Fig 7:**
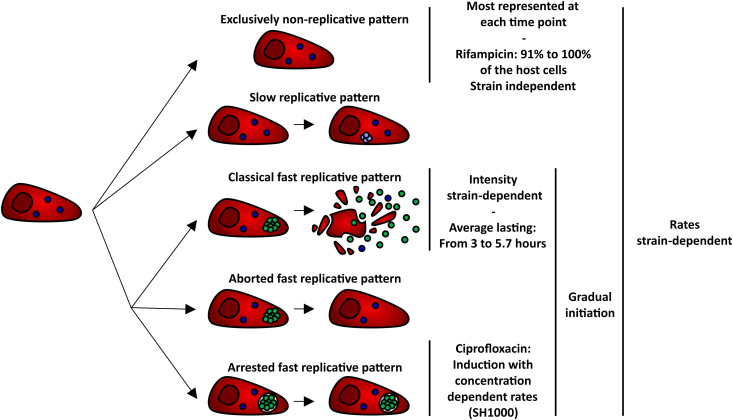
Model of the intracellular *S. aureus* replication dynamics and corresponding antibiotics impact. After *S. aureus* internalization by osteoblastic cells, the entire intracellular bacterial population can remain quiescent, forming the exclusively non-replicative host cell subset. It is the most represented over time for all the tested strains. Within the host cells, at least one *S. aureus* can transition from quiescence to slow or fast replication. This transition occurs gradually over the time course of the infection. Host cells are classified into 3 groups depending on the fast replicative phase pattern: **(i)** “classical” leading to host cell lysis and the subsequent release of intracellular *S. aureus*; **(ii)** “aborted” where the *S. aureus* signal disappears without impacting the host cell phenotype; **(iii)** “arrested” where *S. aureus* experience a growth arrest inside phenotypically unaltered host cell. The rates of transitioning as well as the patterns and intensities of fast replicative phases are strain-dependent. Survival to rifampicin treatment exhibits a population-wide strain-independent predominant or exclusive non-replicative profile. In contrast, ciprofloxacin treatment induces arrested fast replicative phase in a concentration-dependent manner. The combination of both antibiotics does not show superiority compared to rifampicin alone.

Focusing on the intraosteoblastic behavior of *S. aureus* SH1000, we observed that almost half of the infected osteoblasts contained exclusively non-replicative *S. aureus* (Fig 1K). Interestingly, the percentage of host cell carrying exclusively non-replicative bacteria is strain-dependent and could reach more than 80% (Figs 2A and 7). In addition, we highlighted heterogeneity in replicative phases with various kinetics and intensities. These results confirmed the ones obtained by Rodrigues Lopes *et al.* and provide more depth regarding the single cell event [17]. This strain-dependent intracellular replication has also been shown for *Acinetobacter baumannii* using end-point microscopy [47]. As discussed in Rodrigues Lopes *et al.*, the molecular mechanisms associated with the low replication phenotype for the tested clinical strains (S1 Table) are not yet established [17]. Although low-replicative isolates were defective in the agr system, no mutations were identified in the *agr* genes or their promoter [17]. These mutations could have explained the absence of intracellular replication, as it was observed for other strains [48,49] and in this study using the Δ*agrA* SH1000 mutant strain. One hypothesis would be that these strains have a defect in nutrient acquisition, impairing their ability to replicate intracellularly. Indeed, host nutrients are particularly limited intracellularly and mutation in specific metabolic pathways could impact the replication ability of intracellular bacteria [50]. Interestingly, the two clinical isolates ranked in group I as mainly non-replicative were isolated from acute and chronic BJIs stages (S1 Table). Moreover, our ranking of clinical isolates based on their intracellular replicative dynamics matched with the previous study of Rodriguez Lopes *et al.* with minor refinement of two strains (S1 Table) [17].

Then, we challenged infected osteoblasts with rifampicin. Rifampicin is part of the standard-of-care antibiotics for the treatment of BJIs, especially the ones associated with medical devices [51]. This choice is motivated by its strong activity described against biofilm and intracellular staphylococci using CFU method [25,26,52]. However, *in vitro* experiments demonstrated that, even if rifampicin has a strong effect against intracellular bacteria, it cannot totally eradicate the intracellular bacterial inoculum [27,53,54]. Thanks to real time fluorescence imaging, we observed that all infected osteoblasts contained non-replicative *S. aureus* during rifampicin treatment, indicating that only non-replicative *S. aureus* can tolerate antibiotic treatment (Figs 4A and 7). This phenomenon was consistently observed across all the strains tested, supporting a strain-independent phenomenon. Interestingly, we observed that rifampicin-mediated killing happened early in the kinetics and was limited to 0.3 log decrease maximum for all the clinical sensitive strains (S12 Fig). This suggests that rifampicin primarily targets metabolically active and actively replicating bacteria during the early phase of infection. By inhibiting RNA transcription, rifampicin would subsequently induce a dormant-like state in the non-replicative bacteria that renders them tolerant to its own bactericidal activity. Consequently, bacteria that would normally replicate later in the control are unable to do so, having been locked into a non-replicative, dormant-like state. The absence of rifampicin-mediated killing in laboratory strains suggests the rapid induction of rifampicin tolerance in those strains. We showed that the limited effect of rifampicin against intracellular *S. aureus* cannot be attributed to poor penetration by dosing the concentration of rifampicin inside osteoblasts (S9A Fig). We also showed that rifampicin treatment provoked a global decrease of colony size for all the bacterial population, which could be due to the impact of rifampicin on the bacterial RNA pool thus supporting intracellular rifampicin-induced tolerance (Figs 5E and S13). Moreover, we observed high percentages of rifampicin tolerance during killing curves in stationary phases, supporting the idea that rifampicin tolerance is due to the absence of and/or low metabolic activity and replication of *S. aureus* (S8B Fig). These results are consistent with an endpoint flow cytometry study showing intracellular non-replicating *S. aureus* persisters to oxacillin, clarithromycin, and moxifloxacin [35].

Interestingly, intracellular rifampicin efficacy evaluation by CFU did not totally correlate with the replication dynamics of intracellular *S. aureus* (Figs 2A, 4A, 5C and S5). This discrepancy may stem from the temporal differences between CFU assessment and live microscopy. Although strains BJI001 and BJI076 were classified as mainly replicative based on live microscopy, CFU measurements yielded opposite results under rifampicin treatment (Figs 2A and 5C). This may be because, at the time of CFU assessment in the control condition, the fast replication phase of BJI001 had largely concluded, while it was still ongoing for BJI076 thus increasing the number of resulting colonies compared to BJI001 (S7 and S11 Figs). This highlights a limitation of CFU monitoring in evaluating intracellular antibiotic efficacy, as its results can be heavily influenced by the replication dynamics of the bacteria under control conditions.

Our study also highlighted that ciprofloxacin treatment, as rifampicin, even at serum concentration, failed to control the infection, as described for intracellular *Brucella abortus* [55], and induced arrested fast replicative phases in a constrained circular shape suggestive of vacuolar localization (Figs 6 and 7). Ciprofloxacin concentration measurements indicated that the drug likely failed to maintain a high intracellular level (S9B Fig). Concentration below the MIC could impair toxins production or activity, such as phenol soluble modulins required for vacuole escape [56–60]. This may partially explain how ciprofloxacin promoted an arrested fast replicative phase constrained in a circular shape. Additionally, combining ciprofloxacin with rifampicin, considering the specific intracellular focus, presented no additive impact on the infection clearance and the replication dynamics compared to rifampicin alone (Fig 6A). It further supporting the notion that ciprofloxacin contributes little to intracellular bacterial killing under these conditions. This is consistent with the limited clinical efficacy of ciprofloxacin monotherapy and the higher success rates observed when ciprofloxacin is combined with rifampicin, compared to ciprofloxacin alone [30,31,61].

Our study has several limits that could be addressed in further studies. To streamline our screening of different *S. aureus* strains, we used MG-63 osteoblastic cell line, providing consistency and standardization. However, we cannot exclude that these cells present differences with primary bone cells. To confirm the relevance of our results, the replicative dynamics of intracellular *S. aureus* should be further studied in primary osteoblasts. Moreover, other bone cell types such as osteocytes and osteoclasts, have also been identified as hosts for *S. aureus* [13,15]. Future studies should explore bacterial replicative dynamics in these cell types. Additionally, to strengthen the translational significance of our findings, particularly regarding the non-replicative phenotype observed under rifampicin treatment, replication dynamics should be also explored in *ex vivo* or *in vivo* models. Although no established models currently allow direct monitoring of intracellular replication within osteoblasts in bone tissue, our work represents a first step toward developing such approaches in animal models and clinical biopsies. Furthermore, our single-cell live imaging model generates extensive datasets and relies on manual tracking and analysis, which currently limits its throughput. Therefore, it is a complementary tool to fixed-cell studies that can accommodate larger isolate collections.

Another limitation of our study is its focus on the recommended antibiotic treatment consisting of rifampicin and ciprofloxacin. It would be of interest to test other antibiotics relevant to BJIs treatment to investigate whether our findings are generalizable or specific to certain antibiotics. Notably, alternative fluoroquinolones such as ofloxacin and levofloxacin have shown higher intracellular activity *in vitro* [26]. Our current 24-hour observation window is relatively short and technically constrained, but extending it would be valuable. The use of primary cells could help overcome the limitations of MG-63 cell line, whose proliferation and high motility hinder longer-term data analysis. From a more mechanistic point of view, deciphering molecular mechanisms associated with the low intracellular replication phenotype is crucial for our global understanding of antibiotic tolerance as well as identifying potential therapeutic targets to reactivate non-replicative bacteria.

Overall, we highlighted that the replication dynamics of intraosteoblastic *S. aureus* is heterogeneous and that survival of rifampicin treatment is a population-wide phenomenon with a strain-independent non-replicative profile (Fig 7). Osteoblasts could constitute a privileged reservoir for non-replicating *S. aureus*, promoting the chronicization and relapses of BJIs.

## Materials and methods

### Cell line and culture conditions

The MG63 human osteoblastic cell line (Sigma, 86051601), isolated from bone osteosarcoma, was cultured in Dulbecco’s modified Eagle’s medium containing 1 g/L D.glucose and L.glutamine + pyruvate (Gibco, 31885023; termed DMEM) supplemented with 10% fetal bovine serum (Gibco, 42F4572K; termed FBS) and 1% penicillin-streptomycin solution (Gibco, 15140122). Cells were maintained at 37°C in a humidified atmosphere with 5% CO_2_.

### Bacterial strains and clinical isolates

The *Staphylococcus aureus* strains employed in this study are summarized in S1 Table. Clinical isolates of *S. aureus* were selected from a collection of samples obtained from patients suffering a first episode of BJIs at Hospices Civil de Lyon, Lyon, France, from 2001 to 2011 [27]. They were previously characterized phenotypically and are representative of three groups with distinct replicative and host cytotoxicity rates (S1 Table) [17]. All used strains are methicillin-susceptible. They were transformed via electroporation (electroporator settings: 2,5 kV, 100 Ω, 25 µF, exponential) by a pCN47::GFP plasmid carrying an erythromycin resistance gene (*ermC*) and a constitutive *gfp* gene under pblaZ promoter.

### Bacterial mutant construction

The *S. aureus* SH1000 Δ*agrA* mutant was generated by deleting the *agrA* gene using the CRISPR/Cas9 technique with the pCasSA agrA plasmid [62]. Briefly, phage transduction was used to transduce the pCasSA plasmid into the SH1000 strain, as previously described [63]. Genome editing was screened by PCR, then the SH1000 strain Δ*agrA* curated from the plasmid was validated for the absence of off-target by WGS.

### Bacterial culture conditions

All strains and clinical isolates were stored at -80°C in glycerol. Each bacterium was streaked from frozen stock onto Luria-Betani (LB) agar plates supplemented with erythromycin 10 μg/mL (Sigma, E5389) where appropriated, for chromosomally encoded resistance, and incubated aerobically at 37 °C overnight. An isolated colony was then inoculated into LB medium and incubated aerobically at 37 °C overnight at 240 rpm. Where appropriated the medium was supplemented with erythromycin 10 μg/mL.

### Osteoblast and *S. aureus* fluorescent staining

Prior to infection, osteoblasts were washed once with Dulbecco’s Phosphate Buffer Saline free of calcium and magnesium (GIBCO, 14190–094; termed DPBS (-/-)), and labeled with CellTracker Red CMTPX (Thermo Fisher Scientific, C34552) at 10 μM in DMEM without L.glutamine, HEPES and phenol red (Gibco, 11880–028, termed DMEM(-)) during 1h30, protected from light, at 37°C in a humidified atmosphere with 5% CO_2_. Then, cells were washed once with DPBS (-/-) at 37°C to remove any excess dye.

Labelling of *S. aureus* with eBioscience Cell Proliferation Dye eFluor-450 (Thermo Fisher Scientific, 65-0842-85; termed eFluor-450) was performed as previously described for eBioscience Cell Proliferation Dye eFluor-670 with minor modifications [64]. Overnight *S. aureus* cultures were centrifuged at 15,000 rcf for 5 min at 37°C. The pellets were suspended in an equal volume of Dulbecco’s Phosphate Buffer Saline containing calcium and magnesium (Gibco, 14040–091; termed DPBS (+/+)) at 37°C and then pelleted again by centrifugation at 15,000 rcf for 2.5 min at 37°C, repeated twice. The resulting pellets were then suspended in same volume of DPBS (+/+) containing either 10 µM or 25 µM of eFluor-450 at 37°C, for planktonic or infection assays, respectively. Bacterial suspensions were incubated for 5 min at room temperature in the dark and then centrifuged as described. The resulting pellets were suspended in same volume of LB medium at 37°C and incubated at room-temperature for 3 min in the dark to quench any unreacted dye. The bacterial suspensions were washed once with DPBS (+/+) at 37°C, and the resulting pellets were finally suspended in same volume of DPBS (+/+) at 37°C and protected from light. To set up infections at the appropriate multiplicity of infection (MOI), the OD_600nm_ of the resulting labelled *S. aureus* suspensions was adjusted to a defined value for which the concentration of each bacterial strains were known.

### Flow cytometry assay

*S. aureus* SH1000-GFP overnight culture was labelled with eFluor-450, as described above, and diluted in LB medium at 37°C to an OD_600nm_ = 0.05. Bacterial suspension was incubated at 37°C aerobically at 240 rpm. Samples were collected every 30 minutes, except the last time point representing 1-hour gap, and immediately processed by flow cytometry using an Attune NxT Flow Cytometer (Thermo Fisher Scientific). Green and blue fluorescence were measured with a 488 nm and 405 nm laser for excitation and a 530/30 nm or 440/50 nm band pass filter for detection, respectively. *S. aureus* SH1000-GFP strain unlabeled by eFluor-450 served as a negative control. Data were analyzed with FlowJo software (v10.5.3, Treestar Inc.). Forward-scatter area (FSC-A) versus green fluorescence intensity was used to gate out negative GFP-expressing *S. aureus*. Then, damaged or multiplet bacteria were gated out using FSC-A versus forward-scatter heigh (FSC-H) and side-scatter area (SSC-A) versus side-scatter heigh (SSC-H). In the meanwhile, the OD_600nm_ of the samples were monitored using a spectrophotometer. The number of generations, *N*, obtained from both flow cytometry and OD_600nm_ methods were calculated as previously described [35,65] using F = 2^*N*^, F correspond to the ratio Yo/Yt and Y is the mode of the blue fluorescence intensity or the OD_600nm_ value. Doubling times were calculated between the 30-minute and 2-hour time point.

### Minimal inhibitory concentration (MIC) assay

The MICs of rifampicin (Sigma, R3501) were determined by broth microdilution according to the Clinical and Laboratory Standards Institute guidelines (Wayne PA) [66,67]. Briefly, MICs for GFP-expressing *S. aureus* strains and clinical isolates were assessed in LB or DMEM (-) with 10% FBS in 96-wells plate (Falcon, 351172). Inocula were prepared by making a direct saline suspension of isolated colonies from agar plates supplemented with erythromycin 10 μg/mL, adjusted to 0.5 McFarland turbidity, and then diluted in medium to achieve a final concentration of 2.5x10^5^ CFU/mL in each well. Each plate included growth-control and sterility-check wells. The MICs were defined as the lowest concentration of rifampicin that completely inhibited *S. aureus* growth, as determined by the absence of turbidity, measured at 600 nm using a plate reader after 20 hours of incubation at 37°C.

### Time-kills curve (TKC) assay

*S. aureus* SH1000-GFP strain was cultured, as described above, for either 7 hours (exponential phase) or 17 hours (stationary phase). Inoculum suspensions were prepared from the 7-hour culture at 5x10^5^ CFU/mL or directly from the 17-hour culture. These were incubated aerobically at 37°C and treated with a range of rifampicin concentrations (0, 4, 8, 16 and 1000 x MIC). At timed intervals (0, 2, 4, 6, and 24 hours), bacterial cultures were spread on Trypcase Soy Agar plates (Biomérieux, 43011; TSA) and incubated at 37°C for 14 hours to 18 hours. Colonies were then counted using a colony counter (Interscience, Scan4000).

### Mass spectrometry assay

For the mass-spectrometry-based whole-cell rifampicin or ciprofloxacin accumulation assay, MG63 cells were seeded at a confluent density in 24-well plates (Falcon, 353047), one day prior to treatment. Cells were treated with either rifampicin at 6 µg/mL or ciprofloxacin at 2, 5, and 10 µg/mL in DMEM (-) with 10% FBS, or left untreated, and incubated at 37°C in a humidified atmosphere with 5% CO_2_. At 6 hours post-treatment, rifampicin-treated cells were either unwashed or washed with DPBS (-/-) at 37°C and further incubated in DMEM (-) with 10% FBS. Cells were collected at timed intervals of incubation (rifampicin: 5 min, 2 hours, 5 hours, and 18 hours post washing step; ciprofloxacin: 5 min, 1-hour, 6 hours, and 24 hours post-treatment), following washing steps with DPBS (-/-) at 37°C. Collected cells were centrifuged at 490 rcf at 4°C, and the resulting pellets were washed three times with DPBS (-/-) at 4°C. Final pellets were suspended in 50 µL of DPBS (-/-) at 37°C, and transferred to low-binding tubes. Cell concentrations were measured using an automatic cells counter. 50 µL of acetonitrile were added to the cell suspensions, which were incubated for 5 min at room temperature after gentle mixing. Samples were stored at -80°C until mass-spectrometry analysis. Untreated cells were used for standard calibration curves preparation.

Qualitative analysis was performed on a Waters ACQUITY UHPLC HSS T3 column (1.8 μm 50 × 2.1mm) at room temperature with the flow rate of mobile phase set at 0.5 mL/min. The mobile phase consisted of (A) 0.1% formic acid in water, and (B) 0.1% formic acid in 95% acetonitrile, 5% water. A linear gradient elution program was applied as follows: 0–1 min: 0–100% B; 1–1.3 min: 100% B; 1.3-1.4 min: 100%-0% B; 1.4-2.2 min: 0% B. The sample injection volume was 2 μL. Mass spectrometry assays were performed using a TSQ Quantiva mass spectrometer (Thermo Fisher Scientific) interfaced with an UltiMate 3000 XRS UHPLC system (Thermo Fisher Scientific). Data was processed using Trace Finder version 3.3 (Thermo Fisher Scientific). Mass spectrometry detection was performed in positive ion mode and each drug was quantified using one selected reaction monitoring transition (dwell time = 50ms): m/z 823.371 to 791.236 for rifampicin and 332.141 to 287.986 for ciprofloxacin. Collision energy and RF-lens values are detailed in S2 Table.

### Infection of osteoblast cells

MG63 osteoblastic cells were seeded in 6-well plates (Falcon, 353046), 24-well plates (Falcon, 353047; 4TITUDE, 4TI-0241), or 96-well plates (Falcon, 351172; Greiner bio-one, 655090), 24 hours before infection, at 18,000 cells/cm^2^ for sparse density or at 73,000 cells/cm^2^ for confluent density. Prior to infection, MG63 cells were labelled with CellTracker Red CMTPX, where appropriated, as described above. GFP-expressing *S. aureus* overnight cultures were labelled with eFluor-450, unless otherwise stated, and adjusted to an OD_600nm_ corresponding to a known bacterial concentration, as described above. Infections were performed at a MOI of 8, unless otherwise stated, using DMEM (-) with 10% FBS. Following 2 hours of co-incubation at 37°C in a humidified atmosphere with 5% CO_2_, the remaining extracellular *S. aureus* were killed by replacing the medium with DMEM (-) with 10% FBS containing 10 µg/mL lysostaphin (Sigma, L7386) at 37°C, marking the start of the infection timing. Rifampicin and/or ciprofloxacin (Sigma, 17850) were added to the medium where indicated. Lysostaphin and antibiotic treatments were maintained throughout the assay, unless otherwise stated.

### Colony forming unit (CFU) assay

Infection was performed at MOI 8 (or 0.1, 1 10, 100 where indicated) using sparsely seeded, unlabeled MG63 cells, as described above. Rifampicin and/or ciprofloxacin treatments were applied at concentrations of 6 µg/mL and 2 µg/mL, respectively. At 1 hour and 24 hours post-infection (hpi), cells were washed once with DPBS (-/-) and then lysed with H_2_O to collect intracellular *S. aureus*. The resulting cell lysates were diluted as needed and plated on TSA plates using an automatic plate seeder (Interscience, EasySpiral Dilute). Plates were incubated at 37°C for 16h30 and colonies were counted using a colony counter (Interscience, Scan 1200).

### Colony area monitoring

Whole-plate image acquisition was performed using a Scan 1200 colony counter, and colony area quantification was performed using a customized macro in Fiji software (v1.53t, NIH). After edge colonies exclusion, image segmentation was applied using isodata thresholding. Colony areas were then measured with the ‘Analyze Particles’ plugin, using filters for size (0–400) and circularity (0.9-1). SCVs were identified as colonies with an area less than one-fifth of the median area of the untreated control population, following previously established criteria [68].

### Cytotoxicity assay

Infection was performed using confluent, unlabeled cells in 96-well plates (Greiner bio-one, 655090), as described above. Rifampicin treatment was applied at 6 µg/mL, along with 2 µg/mL propidium iodide (Sigma, p4864). At 1 hpi and 24 hpi, red (propidium iodide) fluorescence intensity was measured using a plate reader (Tecan, Spark M10) with a monochromator-fluorescence optics set to 535/20 nm for excitation and 635/20 for detection.

### Live cell imaging

Infection was performed at MOI 8 (or 2 or 4 where indicated) using sparse CellTracker Red CMTPX-labeled cells in 24-well plates (4TITUDE, 4TI-0241) or 96-well plates (Greiner bio-one, 655090), as described above. Rifampicin and/or ciprofloxacin treatment were applied at 6 µg/mL and 2, 5, and 10 µg/mL, respectively. Then, the plates were immediately transferred to the microscope chamber at 37°C in a humidified atmosphere with 5% CO_2_. Time-lapse imaging over 24 hours was conducted with hourly acquisitions using an automated high-content confocal spinning disk (dual wide Nipkow disk) CQ1 microscope (Yokogawa) equipped with a sCMOS camera (2560*2160 pixels) and a 40x objective lens (UPLSAPO, 40x/0.95, WD 0.18 mm, Olympus). Fluorescence from CellTracker Red CMTPX, GFP and eFluor-450 were measured using 561 nm (50 mW, 10%, 250 ms), 488 nm (60 mW, 15%, 200 ms) and 405 nm (100 mW, 10%, 250 ms) lasers for excitation and 617/73 nm, 525/50 nm, or 447/60 nm band pass filters for detection, respectively. At least nine fields per well were imaged.

### Images analysis

Image analysis was performed using a semi-automated macro customized with Fiji software (v1.53t, NIH). For each well, three fields were analyzed. Individual eukaryotic cells were tracked using the ‘TrackMate’ plugin based on red (CellTracker Red CMTPX) fluorescence, excluding edge cells from analysis [69]. At each time point, the number of pixels with green (GFP) and blue (eFluor-450) fluorescence above a fixed threshold, marking *S. aureus*, was recorded for each eukaryotic cell. The resulting data were analyzed with a custom macro in Excel software (v2211, Office, Microsoft). Cells were distinguished as infected and non-infected. Infected cells were further classified into five subsets based on the dynamics of green (GFP) fluorescence pixels, representing *S. aureus* population surface area changes (Figs 1 and 7). (i) Host cells containing exclusively non-replicative *S. aureus*, showing a stable or decreasing green (GFP) pixel count as long as the cell is infected; host cells containing at least one *S. aureus* transitioning to: (ii) slow replicative phase, showing gradual increase in green (GFP) pixels, by 100 or at least 1.33 times, sustained over two or more successive time points; (iii) classical fast replicative phase characterized by a rapid surge in green (GFP) pixels marked by an increase of at least 100 additional pixels at each consecutive time point, sustained over a minimum of two successive intervals, followed by a sharp drop of at least 1.33 times due to host cell lysis; (iv) aborted fast replicative phase, characterized by a rapid surge in green (GFP) pixel, as previously, followed by the disappearance of GFP signal within a single time-point interval, with no effect on host cell phenotype for at least three subsequent time points; (v) arrested fast replicative phase, characterized by a rapid surge in green (GFP) pixel, as previously, followed by stabilized level over at least three successive time points, with no impact on host cell phenotype. Based on this ranking, strains were classified into three groups: group I, comprising strains with a mainly non-replicative profile, defined by more than 80% of host cells harboring exclusively non-replicative *S. aureus* (SH1000 Δ*agrA*, BJI031, and BJI035); group II, representing an intermediate replicative profile, with fewer than 50% of host cells harboring fast replicative *S. aureus* (SH1000, HG001, BJI019, and BJI053); and group III, characterized by a high replication profile, with more than 64% of host cells harboring fast replicative *S. aureus* (BJI076 and BJI001).

The intensity of the classical fast replicative phase, representing the population size increase from replication onset to host cell lysis, was calculated as the ratio of total green (GFP) pixel count within these boundaries. Lysis events occurring during this phase were manually monitored. Co-existing non-replicative *S. aureus*, still labeled with eFluor-450 and sharing host cells with fast replicating *S. aureus*, was also monitored manually. Each cell was visually inspected throughout the kinetics, with data manually controlled and cured.

### Transmission electron microscopy (TEM)

Infection was performed with *S. aureus* SH1000-GFP on confluent, unlabeled cells in 6-well plates (Falcon, 353046), as described above. At 1, 6, and 24 hpi, cells were fixed at room temperature with 2% glutaraldehyde (EMS) in 0.1 M sodium cacodylate (pH 7.4) buffer and washed three times in 0.2 M sodium cacodylate buffer. Cells were post-fixed with 1% aqueous osmium tetroxide (EMS) at room temperature for 1 hour at room temperature, dehydrated through a graded ethanol series and embedded in Epon. Following polymerization, ultrathin sections (100 nm) were cut on a UC7 ultramicrotome (Leica) and collected on 200 mesh grids. Sections were stained with uranyl acetate and lead citrate and imaged using a JEOL 1400JEM transmission electron microscope equipped with an Orius 600 camera and Digital Micrograph.

### Statistical analyses

All statistical analyses were carried out with GraphPad Prism [9.3.1] software (GraphPad Software, Inc., Boston, USA). Mann-Whitney tests were systematically used to compare two groups in order to take into account small sample sizes and/or differences in standard deviation. Multiple comparisons were performed by one-way ANOVA or two-way ANOVA, and post hoc comparisons were corrected using either Dunnett, or Tukey, or Sidak tests. Results were considered significantly different when *p* < 0.05. The results are representative of at least three independent experiments. In cases with technical replica, inter-group and intra-group variances were assessed; given that intra-group variances were consistently higher than inter-group variances, data were treated as independent experiments for analysis.

## Supporting information

S1 Fig The DFD method using eFluor-450 allows monitoring of *S. aureus* replication dynamics for up to 6 generations. **(A-D)** *S. aureus* SH1000 expressing GFP in stationary phase was labeled with eFluor-450 at 10 μM and then diluted in LB medium at 37°C to an initial OD600nm = 0.05. The culture was incubated, and green (GFP) and blue (eFluor-450) fluorescence intensities were analyzed by flow cytometry every 30 minutes, except for the last time point, which was taken at a one-hour interval. OD600nm measurements were taken simultaneously with a spectrophotometer. **(A, B)** Flow cytometry profile representing the frequency of events normalized to mode as a function of blue (eFluor-450) **(A)** or green (GFP) **(B)** intensity over time. It showed either a uniform stepwise halving of blue (eFluor-450) intensity at each time point, indicating a uniform and consistent replication pattern until reaching background noise **(A)** or a maintained green (GFP) intensity, indicating the stability of GFP expression **(B)**. N > 15500 events per time-point were recorded. **(C)** Quantifications from flow cytometry and OD_600nm_ measurements normalized to the last and initial time points, respectively. **(D)** Bacterial replication curves derived from DFD and OD_600nm_ methods normalized by the equation FD_(tn)_ = OD_(tn+1)_ showing similar patterns for 6 generations and doubling times (e.g., 27.6 min for DFD and 31.2 min for OD, calculated between 1 h and 2 h). **(C**, **D)** Results were presented as mean ± SD from 3 independent experiments.
(TIFF)

S2 Fig GFP and eFluor-450 do not impact the invasiveness or intracellular survival of *S. aureus.* MG63 cells, seeded at sparse density and unlabeled, were infected at an MOI of 8 with either *S. aureus* SH1000 or SH1000-GFP strains, pre-labeled or unlabeled with eFluor-450. Following 2 hours of co-incubation, lysostaphin at 10 µg/mL was added to eliminate extracellular *S. aureus*. Intracellular *S. aureus* were collected at 1 and 24 hours post-infection (hpi), and total number of *S. aureus* forming colonies on agar plate was assessed showing no significant differences between conditions at each time-points. Results were presented as mean ± SD from 3 independent experiments in technical triplicate. Two-way analysis of variance (ANOVA) test with Sidak’s correction for multiple comparisons post hoc test (ns: treatment, *p* < 0.0001: time).
(TIFF)

S3 Fig Intracellular *S. aureus* replication dynamics are not impacted by MOI. **(A-C)** MG63 cells, seeded at sparse density and labeled with CellTracker Red CMTPX were infected with *S. aureus* SH1000 expressing GFP strain pre-labeled by eFluor-450 at an MOI of 2, 4, and 8. Following 2 hours of co-incubation, lysostaphin at 10 µg/mL was added to eliminate extracellular *S. aureus*. Time-lapse imaging was conducted over 24 hours with hourly acquisitions using automated confocal microscopy. **(A)** Quantification of the percentage of infected cells. **(B)** Quantification of the *S. aureus* load per cell, measured as GFP pixel count per cell. **(C)** Quantification of infected cells based on the intracellular *S. aureus* replication dynamics (RD) of SH1000 over the 24-hour infection period. **(A-C)** Results were presented as mean ± SD from 3 independent experiments in technical triplicate. One-way ANOVA test with Tukey’s correction for multiple comparisons post hoc test: *p < 0.05, ****p < 0.0001.
(TIFF)

S4 Fig Invasion and intracellular survival of *S. aureus* are strain-dependent. MG63 cells, seeded at confluent density and unlabeled, were infected with a range of *S. aureus* strains and clinical isolates expressing GFP at MOI 8 pre-labeled by eFluor-450 (S1 Table). Following 2 hours of co-incubation, lysostaphin at 10 µg/mL was added to eliminate extracellular *S. aureus*. Intracellular *S. aureus* were collected at 1 hpi and 24 hpi, and the total number of *S. aureus* forming colonies on agar plate was investigated. Total number of intracellular *S. aureus* SH1000 forming colonies on plates. Results were presented as mean ± SD representing 12 individual values from 4 independent experiments. Mann-Whitney test: *p < 0.05, **p < 0.01, ****p < 0.0001.
(TIFF)

S5 Fig Replication dynamics is heterogeneous among strains. MG63 cells, seeded at sparse density and labeled with CellTracker Red CMTPX, were infected with a range of *S. aureus* strains and clinical isolates expressing GFP at MOI 8 pre-labeled by eFluor-450 (S1 Table). Following 2 hours of co-incubation, lysostaphin at 10 µg/mL was added to eliminate extracellular *S. aureus*. Concomitantly, rifampicin was added or not at 6 µg/mL. Time-lapse imaging was conducted over 24 hours with hourly acquisitions using automated confocal microscopy. Quantification of infected cells based on intracellular *S. aureus* replication dynamics (RD) performed hourly across the 24-hour infection period. Results were presented as mean ± SD from 3 independent experiments in technical triplicate.
(TIFF)

S6 Fig Absence of replication prevents cytotoxicity. MG63 cells, seeded at confluent density and unlabeled, were infected with a range of *S. aureus* strains and clinical isolates expressing GFP at MOI 8 (S1 Table). Following 2 hours of co-incubation, lysostaphin at 10 µg/mL was added, to eliminate extracellular *S. aureus*. Concomitantly, propidium iodide (PI) was added at 2 µg/mL. At 24 hpi PI fluorescence intensity was measured with a plate reader. PI signal indicates *S. aureus*-induced cytotoxicity trough host cells membrane damage. Results were presented as mean ± SD representing 9 individual values from 3 independent experiments. One-way ANOVA with Dunnett’s correction for multiple post hoc comparisons with the control and Mann-Whitney test: **p < 0.01, ****p < 0.0001.
(TIFF)

S7 Fig Transitioning from quiescence to fast replicative phase is gradual among strains. MG63 cells, seeded at sparse density and labeled with CellTracker Red CMTPX, were infected with a range of *S. aureus* strains and clinical isolates expressing GFP at MOI 8 pre-labeled by eFluor-450 (S1 Table). Following 2 hours of co-incubation, lysostaphin at 10 µg/mL was added to eliminate extracellular *S. aureus*. Concomitantly, rifampicin was added or not at 6 µg/mL. Time-lapse imaging was conducted over 24 hours with hourly acquisitions using automated confocal microscopy. Quantification of the initiation and duration of the fast replicative phase for each strain, with ongoing fast replicative phases at the end of the observation period excluded from analysis. Results were presented as median and quartiles from 3 independent experiments in technical triplicate. (ND = Not detected).
(TIFF)

S8 Fig Time-kill curves of planktonic *S. aureus* cultures treated with rifampicin. *S. aureus* SH1000 expressing GFP was cultivated in LB medium to reach either the exponential phase **(A)** or stationary phase **(B)**. Cultures were subsequently treated with a range of concentrations of rifampicin (0; 4; 8; 16; 1000; and 10,000 times the MIC). Samples were collected at designated intervals, diluted and plated on agar plates to quantify CFU. Total number of *S. aureus* forming colonies was investigated displaying differences in bacterial survival across concentrations and growth phases. Results represent a single experiment.
(TIFF)

S9 Fig Intracellular accumulation of rifampicin and ciprofloxacin. **(A, B)** MG63 cells, seeded at confluent density and unlabeled, were treated either with rifampicin at 6 µg/mL or ciprofloxacin at 2, 5, and 10 µg/mL. For rifampicin, cells were either washed or left unwashed 6 hours post-treatment, followed by collection after 5 minutes, 2 hours, 5 hours, and 18 hours. For ciprofloxacin, cells were collected at 5 minutes, 1 hour, 6 hours, and 24 hours post-treatment. Intracellular concentrations of rifampicin **(A)** and ciprofloxacin **(B)** were measured using mass spectrometry. Results were presented as mean ± SD from 2 independent experiments.
(TIFF)

S10 Fig Rifampicin survivors exhibit a strain independent, predominant or exclusive non-replicative profile. MG63 cells, seeded at sparse density and labeled with CellTracker Red CMTPX, were infected with a range of *S. aureus* strains and clinical isolates expressing GFP at MOI 8 pre-labeled by eFluor-450 (S1 Table). Following 2 hours of co-incubation, lysostaphin at 10 µg/mL was added to eliminate extracellular *S. aureus*. Concomitantly, rifampicin was added or not at 6 µg/mL. Time-lapse imaging was conducted over 24 hours with hourly acquisitions using automated confocal microscopy. Quantification of infected cells based on intracellular *S. aureus* replication dynamics (RD) performed hourly across the 24-hour infection period. Results were presented as mean ± SD from 3 independent experiments in technical triplicate.
(TIFF)

S11 Fig Rifampicin erases intracellular *S. aureus* replication and survivors are non-replicative. MG63 cells, seeded at sparse density and labeled with CellTracker Red CMTPX, were infected with a range of *S. aureus* strains and clinical isolates expressing GFP at MOI 8 pre-labeled by eFluor-450 (S1 Table). Following 2 hours of co-incubation, lysostaphin at 10 µg/mL was added to eliminate extracellular *S. aureus*. Concomitantly, rifampicin was added or not at 6 µg/mL. Time-lapse imaging was conducted over 24 hours with hourly acquisitions using automated confocal microscopy. Quantification of the *S. aureus* population size per cell over time represented by the total green (GFP) pixel count normalized per cell. Results were presented as individuals or mean ± SD from 3 independent experiments in technical triplicate.
(TIFF)

S12 Fig Rifampicin action is limited in time and rates. MG63 cells, seeded at sparse density and labeled with CellTracker Red CMTPX, were infected with a range of *S. aureus* strains and clinical isolates expressing GFP at MOI 8 pre-labeled by eFluor-450 (S1 Table). Following 2 hours of co-incubation, lysostaphin at 10 µg/mL was added to eliminate extracellular *S. aureus*. Concomitantly, rifampicin was added or not at 6 µg/mL. Time-lapse imaging was conducted over 24 hours with hourly acquisitions using automated confocal microscopy. Quantification of the *S. aureus* population size, from the exclusively non-replicative osteoblasts subset, over time represented by the total green (GFP) pixel count in the control (black curves) and the rifampicin-treated condition (blue curves). Rifampicin action was calculated through the difference between the control and rifampicin treated conditions (orange curves). Cumulative quantification of infected cells in the control condition experiencing a fast replicative phase (green curves). Results were presented as mean ± SD from 3 independent experiments in technical triplicate.
(TIFF)

S13 Fig Effect of rifampicin and/or ciprofloxacin treatments on SCV rates and colony area distribution. MG63 cells, seeded at sparse density and labeled with CellTracker Red CMTPX, were infected with a range of *S. aureus* strains and clinical isolates expressing GFP at MOI 8 pre-labeled by eFluor-450 (S1 Table). Following 2 hours of co-incubation, lysostaphin at 10 µg/mL was added to eliminate extracellular *S. aureus*. Concomitantly, rifampicin and/or ciprofloxacin were added or not at 6 µg/mL and 2 µg/mL, respectively. Intracellular *S. aureus* were collected at 1 hpi and 24 hpi, and the total number of *S. aureus* forming colonies on agar plate was investigated. **(A)** Quantification of the small colony variant (SCV) phenotype, identified as colonies with an area less than 1/5th of the median area of control condition. **(B, C)** Distribution frequency of colony area of collected intracellular *S. aureus* at 1 hpi **(B)** or 24 hpi **(C)**. **(A-C)** Results were presented as mean ± SD representing 9 individual values **(A)** from 3 independent experiments **(A-C)**. Two-way ANOVA test with Sidak’s correction for multiple comparisons post hoc test (*p* < 0.0001: treatment, *p* < 0.0001: time): **p < 0.01, ****p < 0.0001.
(TIFF)

S14 Fig Long-term intracellular *S. aureus* survivors reside principally inside single membrane vacuoles. **(A-H)** Unlabeled MG63 cells were seeded at sparse density and infected at MOI 8 with *S. aureus* SH1000 expressing GFP pre-labeled by eFluor-450. Following 2 hours of co-incubation, lysostaphin at 10 µg/mL was added to eliminate extracellular *S. aureus*. Concomitantly, cells were left untreated **(A**-**F)** or treated with rifampicin at 6 µg/mL **(G**, **H)**. At 24 hpi cells were fixed and imaged by transmission electron microscopy (TEM). (**A-H)** Representative TEM images showing intracellular *S. aureus* residing either within a multilamellar (**A**, **B**; black arrow) or a single-membrane vacuole (**C-E**, **G**, **H**; black arrow) or in the cytoplasm (**F**; white arrow). The intra-vacuolar space is either clear (**D**, **H**; black arrowhead) or dense (**E**; white arrowhead) to the electrons (scale bar = 0.4 µm) **I, J** Corresponding microscopy quantification of events where isolated or small cluster of 2–3 *S. aureus* localized within vacuoles **(I)** and events where the vacuoles are tightly associated with the bacteria **(J)**. Results were presented as mean ± SD representing 3 individual values from 3 independent experiments. Control = 124 events, 179 bacteria; rifampicin = 126 events, 172 bacteria. Mann-Whitney test **(I)** or chi-square test **(J)**: **p < 0.01.
(TIFF)

S1 Table List of the *S. aureus* strains/clinical isolates employed in this study. (TIFF)

S2 Table Mass spectrometry collision energy and RF-lens values. (TIFF)

S1 Movie Exclusively non-replicative host cell subset. Osteoblast (Red) infected by *S. aureus* SH1000 expressing GFP strain labeled with eFluor-450 (GFP: Green and eFluor-450: Blue) showing exclusively non-replicative profile. Scale bar = 10 µm.
(AVI)

S2 Movie Slow replicative host cell subset. Osteoblast (Red) infected by *S. aureus* SH1000 expressing GFP strain labeled with eFluor-450 (GFP: Green and eFluor-450: Blue) showing slow replicative profile. Scale bar = 10 µm.
(AVI)

S3 Movie Fast replicative host cell subset. Osteoblast (Red) infected by *S. aureus* SH1000 expressing GFP strain labeled with eFluor-450 (GFP: Green and eFluor-450: Blue) showing fast replicative profile. Scale bar = 10 µm.
(AVI)

S4 Movie Classical fast replicative phase in an irregular shape. Osteoblast (Red) infected by *S. aureus* HG001 expressing GFP strain labeled with eFluor-450 (GFP: Green and eFluor-450: Blue) showing classical fast replicative phase in an irregular shape. Scale bar = 10 µm.
(AVI)

S5 Movie Classical fast replicative phase constrained in a regular shape. Osteoblast (Red) infected by *S. aureus* HG001 expressing GFP strain labeled with eFluor-450 (GFP: Green and eFluor-450: Blue) showing classical fast replicative phase constrained in a regular shape. Scale bar = 10 µm.
(AVI)

S6 Movie Aborted fast replicative host cell subset. Osteoblast (Red) infected by *S. aureus* BJI001 expressing GFP strain labeled with eFluor-450 (GFP: Green and eFluor-450: Blue) showing aborted fast replicative profile. Scale bar = 10 µm.
(AVI)

S7 Movie Arrested fast replicative host cell subset. Osteoblast (Red) infected by *S. aureus* BJI019 expressing GFP strain labeled with eFluor-450 (GFP: Green and eFluor-450: Blue) showing arrested fast replicative profile. Scale bar = 10 µm.
(AVI)

S8 Movie Field of view of control condition. Osteoblast (Red) infected by *S. aureus* BJI076 expressing GFP strain labeled with eFluor-450 (GFP: Green and eFluor-450: Blue) showing exclusively non-replicative, slow replicative, and fast replicative profiles. Scale bar = 100 µm.
(7Z)

S9 Movie Field of view of rifampicin treated condition. Osteoblast (Red) infected by *S. aureus* BJI076 expressing GFP strain labeled with eFluor-450 (GFP: Green and eFluor-450: Blue) showing exclusively non-replicative profiles. Scale bar = 100 µm.
(7Z)

S10 Movie Arrested fast replicative phase induced by ciprofloxacin treatment at clinical bone concentration. Osteoblast (Red) infected by *S. aureus* SH1000 expressing GFP strain labeled with eFluor-450 (GFP: Green and eFluor-450: Blue) showing arrested fast replicative profile. Scale bar = 10 µm.
(AVI)

S1 Data Primary quantitative data. Collated and indexed quantitative data that underpins all figure of this study.
(XLSX)

## References

[1] Tong SYC, Davis JS, Eichenberger E, Holland TL, Fowler VG Jr. Staphylococcus aureus infections: epidemiology, pathophysiology, clinical manifestations, and management. Clin Microbiol Rev. 2015;28(3):603–61. doi: 10.1128/CMR.00134-14 26016486 PMC4451395

[2] Lowy FD. Staphylococcus aureus infections. N Engl J Med. 1998;339(8):520–32. doi: 10.1056/NEJM199808203390806 9709046

[3] Keim KC, Horswill AR. Staphylococcus aureus. Trends Microbiol. 2023;31(12):1300–1. doi: 10.1016/j.tim.2023.07.001 37487767

[4] Howden BP, Giulieri SG, Wong Fok Lung T, Baines SL, Sharkey LK, Lee JYH, et al. Staphylococcus aureus host interactions and adaptation. Nat Rev Microbiol. 2023;21(6):380–95. doi: 10.1038/s41579-023-00852-y 36707725 PMC9882747

[5] Masters EA, Ricciardi BF, Bentley KL de M, Moriarty TF, Schwarz EM, Muthukrishnan G. Skeletal infections: microbial pathogenesis, immunity and clinical management. Nat Rev Microbiol. 2022;20(7):385–400. doi: 10.1038/s41579-022-00686-0 35169289 PMC8852989

[6] Valour F, Bouaziz A, Karsenty J, Ader F, Lustig S, Laurent F, et al. Determinants of methicillin-susceptible Staphylococcus aureus native bone and joint infection treatment failure: a retrospective cohort study. BMC Infect Dis. 2014;14:443. doi: 10.1186/1471-2334-14-443 25128919 PMC4147168

[7] Wieland BW, Marcantoni JR, Bommarito KM, Warren DK, Marschall J. A retrospective comparison of ceftriaxone versus oxacillin for osteoarticular infections due to methicillin-susceptible Staphylococcus aureus. Clin Infect Dis. 2012;54:585–90. 22144536 10.1093/cid/cir857 PMC3275755

[8] Garcia del Pozo E, Collazos J, Carton JA, Camporro D, Asensi V. Factors predictive of relapse in adult bacterial osteomyelitis of long bones. BMC Infect Dis. 2018;18:635. 30526540 10.1186/s12879-018-3550-6 PMC6286499

[9] Surewaard BGJ, Deniset JF, Zemp FJ, Amrein M, Otto M, Conly J, et al. Identification and treatment of the Staphylococcus aureus reservoir in vivo. J Exp Med. 2016;213(7):1141–51. doi: 10.1084/jem.20160334 27325887 PMC4925027

[10] Hommes JW, Surewaard BGJ. Intracellular Habitation of Staphylococcus aureus: Molecular Mechanisms and Prospects for Antimicrobial Therapy. Biomedicines. 2022;10(8):1804. doi: 10.3390/biomedicines10081804 36009351 PMC9405036

[11] Hudson MC, Ramp WK, Nicholson NC, Williams AS, Nousiainen MT. Internalization of Staphylococcus aureus by cultured osteoblasts. Microb Pathog. 1995;19(6):409–19. doi: 10.1006/mpat.1995.0075 8852281

[12] Josse J, Velard F, Gangloff SC. Staphylococcus aureus vs. osteoblast: relationship and consequences in osteomyelitis. Front Cell Infect Microbiol. 2015;5:85. 26636047 10.3389/fcimb.2015.00085 PMC4660271

[13] Krauss JL, et al. Staphylococcus aureus infects osteoclasts and replicates intracellularly. mBio. 2019;10:e02447–19. 10.1128/mBio.02447-19 PMC6794488 31615966

[14] Garcia-Moreno M, Jordan PM, Günther K, Dau T, Fritzsch C, Vermes M, et al. Osteocytes Serve as a Reservoir for Intracellular Persisting Staphylococcus aureus Due to the Lack of Defense Mechanisms. Front Microbiol. 2022;13:937466. doi: 10.3389/fmicb.2022.937466 35935196 PMC9355688

[15] Yang D, et al. Novel insights into Staphylococcus aureus deep bone infections: the involvement of osteocytes. mBio. 2018;9:e00415–18. 10.1128/mBio.00415-18 PMC5915738 29691335

[16] Walter N, Mendelsohn D, Brochhausen C, Rupp M, Alt V. Intracellular S. aureus in osteoblasts in a clinical sample from a patient with chronic osteomyelitis—A case report. Pathogens. 2021;10:1064. 34451528 10.3390/pathogens10081064 PMC8401219

[17] Rodrigues Lopes I, Alcantara LM, Silva RJ, Josse J, Vega EP, Cabrerizo AM, et al. Microscopy-based phenotypic profiling of infection by Staphylococcus aureus clinical isolates reveals intracellular lifestyle as a prevalent feature. Nat Commun. 2022;13(1):7174. doi: 10.1038/s41467-022-34790-9 36418309 PMC9684519

[18] Rollin G, et al. Intracellular survival of Staphylococcus aureus in endothelial cells: A matter of growth or persistence. Front Microbiol. 2017;8:1354. 28769913 10.3389/fmicb.2017.01354 PMC5515828

[19] Palma Medina LM, Becker A-K, Michalik S, Yedavally H, Raineri EJM, Hildebrandt P, et al. Metabolic Cross-talk Between Human Bronchial Epithelial Cells and Internalized Staphylococcus aureus as a Driver for Infection. Mol Cell Proteomics. 2019;18(5):892–908. doi: 10.1074/mcp.RA118.001138 30808728 PMC6495256

[20] Flannagan RS, Heit B, Heinrichs DE. Intracellular replication of Staphylococcus aureus in mature phagolysosomes in macrophages precedes host cell death, and bacterial escape and dissemination: S. aureus replicates in mature phagolysosomes in macrophages. Cell Microbiol. 2016;18:514–35. 26408990 10.1111/cmi.12527

[21] Kahl BC, Goulian M, van Wamel W, Herrmann M, Simon SM, Kaplan G, et al. Staphylococcus aureus RN6390 replicates and induces apoptosis in a pulmonary epithelial cell line. Infect Immun. 2000;68(9):5385–92. doi: 10.1128/IAI.68.9.5385-5392.2000 10948168 PMC101802

[22] Jubrail J, Morris P, Bewley MA, Stoneham S, Johnston SA, Foster SJ, et al. Inability to sustain intraphagolysosomal killing of Staphylococcus aureus predisposes to bacterial persistence in macrophages. Cell Microbiol. 2016;18(1):80–96. doi: 10.1111/cmi.12485 26248337 PMC4778410

[23] Melly MA, Thomison JB, Rogers DE. Fate of staphylococci within human leukocytes. J Exp Med. 1960;112(6):1121–30. doi: 10.1084/jem.112.6.1121 13769271 PMC2137327

[24] Rogers DE, Tompsett R. The survival of staphylococci within human leukocytes. J Exp Med. 1952;95(2):209–30. doi: 10.1084/jem.95.2.209 14907971 PMC2212057

[25] Marro FC, Abad L, Blocker AJ, Laurent F, Josse J, Valour F. In vitro antibiotic activity against intraosteoblastic Staphylococcus aureus: a narrative review of the literature. J Antimicrob Chemother. 2021;76(12):3091–102. doi: 10.1093/jac/dkab301 34459881 PMC8598303

[26] Zelmer AR, Nelson R, Richter K, Atkins GJ. Can intracellular Staphylococcus aureus in osteomyelitis be treated using current antibiotics? A systematic review and narrative synthesis. Bone Res. 2022;10(1):53. doi: 10.1038/s41413-022-00227-8 35961964 PMC9374758

[27] Valour F, Trouillet-Assant S, Riffard N, Tasse J, Flammier S, Rasigade J-P, et al. Antimicrobial activity against intraosteoblastic Staphylococcus aureus. Antimicrob Agents Chemother. 2015;59(4):2029–36. doi: 10.1128/AAC.04359-14 25605365 PMC4356812

[28] Abad L, et al. Lysosomal alkalization to potentiate eradication of intra-osteoblastic Staphylococcus aureus in the bone and joint infection setting. Clin Microbiol Infect. 2022;28:135.e1–e7. 10.1016/j.cmi.2021.04.030 33962064

[29] Meléndez-Carmona MÁ, Muñoz-Gallego I, Viedma E, Lora-Tamayo J, Chaves F. Intraosteoblastic activity of levofloxacin and rifampin alone and in combination against clinical isolates of meticillin-susceptible Staphylococcus aureus causing prosthetic joint infection. Int J Antimicrob Agents. 2019;54(3):356–60. doi: 10.1016/j.ijantimicag.2019.06.018 31254616

[30] Spellberg B, Lipsky BA. Systemic antibiotic therapy for chronic osteomyelitis in adults. Clin Infect Dis. 2012;54:393–407. 22157324 10.1093/cid/cir842 PMC3491855

[31] Berdal J-E, Skråmm I, Mowinckel P, Gulbrandsen P, Bjørnholt JV. Use of rifampicin and ciprofloxacin combination therapy after surgical debridement in the treatment of early manifestation prosthetic joint infections. Clin Microbiol Infect. 2005;11(10):843–5. doi: 10.1111/j.1469-0691.2005.01230.x 16153261

[32] Osmon DR, Berbari EF, Berendt AR, Lew D, Zimmerli W, Steckelberg JM, et al. Diagnosis and management of prosthetic joint infection: clinical practice guidelines by the Infectious Diseases Society of America. Clin Infect Dis. 2013;56(1):e1–25. doi: 10.1093/cid/cis803 23223583

[33] Bigger J. Treatment of staphylococal infections with penicillin by intermittent sterilisation. Lancet. 1944;244:497–500.

[34] Balaban NQ, Helaine S, Lewis K, Ackermann M, Aldridge B, Andersson DI, et al. Definitions and guidelines for research on antibiotic persistence. Nat Rev Microbiol. 2019;17(7):441–8. doi: 10.1038/s41579-019-0196-3 30980069 PMC7136161

[35] Peyrusson F, et al. Intracellular Staphylococcus aureus persisters upon antibiotic exposure. Nat Commun. 2020;11:2200. 32366839 10.1038/s41467-020-15966-7 PMC7198484

[36] Kerr MC, Wang JTH, Castro NA, Hamilton NA, Town L, Brown DL, et al. Inhibition of the PtdIns(5) kinase PIKfyve disrupts intracellular replication of Salmonella. EMBO J. 2010;29(8):1331–47. doi: 10.1038/emboj.2010.28 20300065 PMC2868569

[37] Malik-Kale P, Winfree S, Steele-Mortimer O. The bimodal lifestyle of intracellular Salmonella in epithelial cells: replication in the cytosol obscures defects in vacuolar replication. PLoS One. 2012;7(6):e38732. doi: 10.1371/journal.pone.0038732 22719929 PMC3374820

[38] Marro FC, Laurent F, Josse J, Blocker AJ. Methods to monitor bacterial growth and replicative rates at the single-cell level. FEMS Microbiol Rev. 2022;30. 10.1093/femsre/fuac030 PMC9629498 35772001

[39] Dyon-Tafani V, Josse J, Dieppois G, Ferry T, Laurent F. Antimicrobial activity of the new FabI inhibitor afabicin desphosphono against intraosteoblastic Staphylococcus aureus. Int J Antimicrob Agents. 2021;57(5):106321. doi: 10.1016/j.ijantimicag.2021.106321 33716179

[40] Proctor RA, von Eiff C, Kahl BC, Becker K, McNamara P, Herrmann M, et al. Small colony variants: a pathogenic form of bacteria that facilitates persistent and recurrent infections. Nat Rev Microbiol. 2006;4(4):295–305. doi: 10.1038/nrmicro1384 16541137

[41] Proctor RA, Kriegeskorte A, Kahl BC, Becker K, Löffler B, Peters G. Staphylococcus aureus Small Colony Variants (SCVs): a road map for the metabolic pathways involved in persistent infections. Front Cell Infect Microbiol. 2014;4:99. doi: 10.3389/fcimb.2014.00099 25120957 PMC4112797

[42] Loss G, Simões PM, Valour F, Cortês MF, Gonzaga L, Bergot M, et al. Staphylococcus aureus Small Colony Variants (SCVs): News From a Chronic Prosthetic Joint Infection. Front Cell Infect Microbiol. 2019;9:363. doi: 10.3389/fcimb.2019.00363 31696062 PMC6817495

[43] Sendi P, Rohrbach M, Graber P, Frei R, Ochsner PE, Zimmerli W. Staphylococcus aureus small colony variants in prosthetic joint infection. Clin Infect Dis. 2006;43(8):961–7. doi: 10.1086/507633 16983605

[44] Lemaignen A, Bernard L, Marmor S, Ferry T, Grammatico-Guillon L, Astagneau P, et al. Epidemiology of complex bone and joint infections in France using a national registry: The CRIOAc network. J Infect. 2021;82(2):199–206. doi: 10.1016/j.jinf.2020.12.010 33352213

[45] Deny A, Loiez C, Deken V, Putman S, Duhamel A, Girard J, et al. Epidemiology of patients with MSSA versus MRSA infections of orthopedic implants: Retrospective study of 115 patients. Orthop Traumatol Surg Res. 2016;102(7):919–23. doi: 10.1016/j.otsr.2016.08.012 27744001

[46] Alder KD, Lee I, Munger AM, Kwon H-K, Morris MT, Cahill SV, et al. Intracellular Staphylococcus aureus in bone and joint infections: A mechanism of disease recurrence, inflammation, and bone and cartilage destruction. Bone. 2020;141:115568. doi: 10.1016/j.bone.2020.115568 32745687

[47] Rubio T, et al. Incidence of an intracellular multiplication niche among Acinetobacter baumannii clinical isolates. mSystems. 2022;7:e0048821. 10.1128/msystems.00488-21 PMC8805633 35103489

[48] Hachani A, Giulieri SG, Guérillot R, Walsh CJ, Herisse M, Soe YM, et al. A high-throughput cytotoxicity screening platform reveals agr-independent mutations in bacteraemia-associated Staphylococcus aureus that promote intracellular persistence. Elife. 2023;12:e84778. doi: 10.7554/eLife.84778 37289634 PMC10259494

[49] Qazi SNA, et al. agr Expression Precedes Escape of Internalized Staphylococcus aureus from the Host Endosome. Infect Immun. 2001;69:7074–82. 11598083 10.1128/IAI.69.11.7074-7082.2001 PMC100088

[50] Best A, Abu Kwaik Y. Nutrition and Bipartite Metabolism of Intracellular Pathogens. Trends Microbiol. 2019;27(6):550–61. doi: 10.1016/j.tim.2018.12.012 30655036 PMC6527459

[51] Zimmerli W, Waldvogel FA, Vaudaux P, Nydegger UE. Pathogenesis of foreign body infection: description and characteristics of an animal model. J Infect Dis. 1982;146(4):487–97. doi: 10.1093/infdis/146.4.487 7119479

[52] Renz N, Trampuz A, Zimmerli W. Controversy about the role of rifampin in biofilm infections: is it justified? Antibiotics. 2021;10:165. 33562821 10.3390/antibiotics10020165 PMC7916064

[53] Abad L, Josse J, Tasse J, Lustig S, Ferry T, Diot A, et al. Antibiofilm and intraosteoblastic activities of rifamycins against Staphylococcus aureus: promising in vitro profile of rifabutin. J Antimicrob Chemother. 2020;75(6):1466–73. doi: 10.1093/jac/dkaa061 32125419

[54] Ellington JK, et al. Intracellular Staphylococcus aureus and antibiotic resistance: implications for treatment of staphylococcal osteomyelitis. J Orthop Res Off Publ Orthop Res Soc. 2006;24:87–93. 10.1002/jor.20003 16419973

[55] Mode S, Ketterer M, Québatte M, Dehio C. Antibiotic persistence of intracellular Brucella abortus. PLoS Negl Trop Dis. 2022;16(7):e0010635. doi: 10.1371/journal.pntd.0010635 35881641 PMC9355222

[56] Schnaith A, Kashkar H, Leggio SA, Addicks K, Krönke M, Krut O. Staphylococcus aureus subvert autophagy for induction of caspase-independent host cell death. J Biol Chem. 2007;282(4):2695–706. doi: 10.1074/jbc.M609784200 17135247

[57] Giese B, Glowinski F, Paprotka K, Dittmann S, Steiner T, Sinha B, et al. Expression of δ-toxin by Staphylococcus aureus mediates escape from phago-endosomes of human epithelial and endothelial cells in the presence of β-toxin. Cell Microbiol. 2011;13(2):316–29. doi: 10.1111/j.1462-5822.2010.01538.x 20946243

[58] Grosz M, Kolter J, Paprotka K, Winkler A-C, Schäfer D, Chatterjee SS, et al. Cytoplasmic replication of Staphylococcus aureus upon phagosomal escape triggered by phenol-soluble modulin α. Cell Microbiol. 2014;16(4):451–65. doi: 10.1111/cmi.12233 24164701 PMC3969633

[59] Strobel M, et al. Post-invasion events after infection with Staphylococcus aureus are strongly dependent on both the host cell type and the infecting S. aureus strain. Clin Microbiol Infect Off Publ Eur Soc Clin Microbiol Infect Dis. 2016;22;799–809. 10.1016/j.cmi.2016.06.020 27393124

[60] Andersson DI, Hughes D. Microbiological effects of sublethal levels of antibiotics. Nat Rev Microbiol. 2014;12(7):465–78. doi: 10.1038/nrmicro3270 24861036

[61] Haddad N, Ajaz J, Mansour L, Kasemodel R, Jarvis J, Jarad J, et al. A Review of the Clinical Utilization of Oral Antibacterial Therapy in the Treatment of Bone Infections in Adults. Antibiotics (Basel). 2023;13(1):4. doi: 10.3390/antibiotics13010004 38275315 PMC10812599

[62] Chen W, Zhang Y, Yeo WS, Bae T, Ji Q. Rapid and efficient genome editing in Staphylococcus aureus by using an engineered CRISPR/Cas9 system. J Am Chem Soc. 2017;139:3790–5. 28218837 10.1021/jacs.6b13317

[63] Olson ME. Bacteriophage Transduction in Staphylococcus aureus. Methods Mol Biol. 2016;1373:69–74. doi: 10.1007/7651_2014_186 25646608

[64] Flannagan RS, Heinrichs DE. A Fluorescence Based-Proliferation Assay for the Identification of Replicating Bacteria Within Host Cells. Front Microbiol. 2018;9:3084. doi: 10.3389/fmicb.2018.03084 30619165 PMC6299164

[65] Helaine S, Thompson JA, Watson KG, Liu M, Boyle C, Holden DW. Dynamics of intracellular bacterial replication at the single cell level. Proc Natl Acad Sci U S A. 2010;107(8):3746–51. doi: 10.1073/pnas.1000041107 20133586 PMC2840444

[66] CLSI. Methods for Dilution Antimicrobial Susceptibility Tests for Bacteria That Grow Aerobically. 11th ed. CLSI standard M07. Wayne (PA): Clinical and Laboratory Standards Institute; 2018.

[67] CLSI. Performance Standards for Antimicrobial Susceptibility Testing. 29th ed. CLSI supplement M100. Wayne (PA): Clinical and Laboratory Standards Institute; 2019.

[68] Tuchscherr L, Medina E, Hussain M, Völker W, Heitmann V, Niemann S, et al. Staphylococcus aureus phenotype switching: an effective bacterial strategy to escape host immune response and establish a chronic infection. EMBO Mol Med. 2011;3(3):129–41. doi: 10.1002/emmm.201000115 21268281 PMC3395110

[69] Tinevez J-Y, Perry N, Schindelin J, Hoopes GM, Reynolds GD, Laplantine E, et al. TrackMate: An open and extensible platform for single-particle tracking. Methods. 2017;115:80–90. doi: 10.1016/j.ymeth.2016.09.016 27713081

