# Supplementary figures and images for: Monitoring intracellular replication dynamics unveils high proportion of non-replicating antibiotic-tolerant Staphylococcus aureus inside osteoblasts
Source: PLoS Pathog. 2025 Sep 23;21(9):e1013525. doi: 10.1371/journal.ppat.1013525 (PMC12478902; doi:10.1371/journal.ppat.1013525)

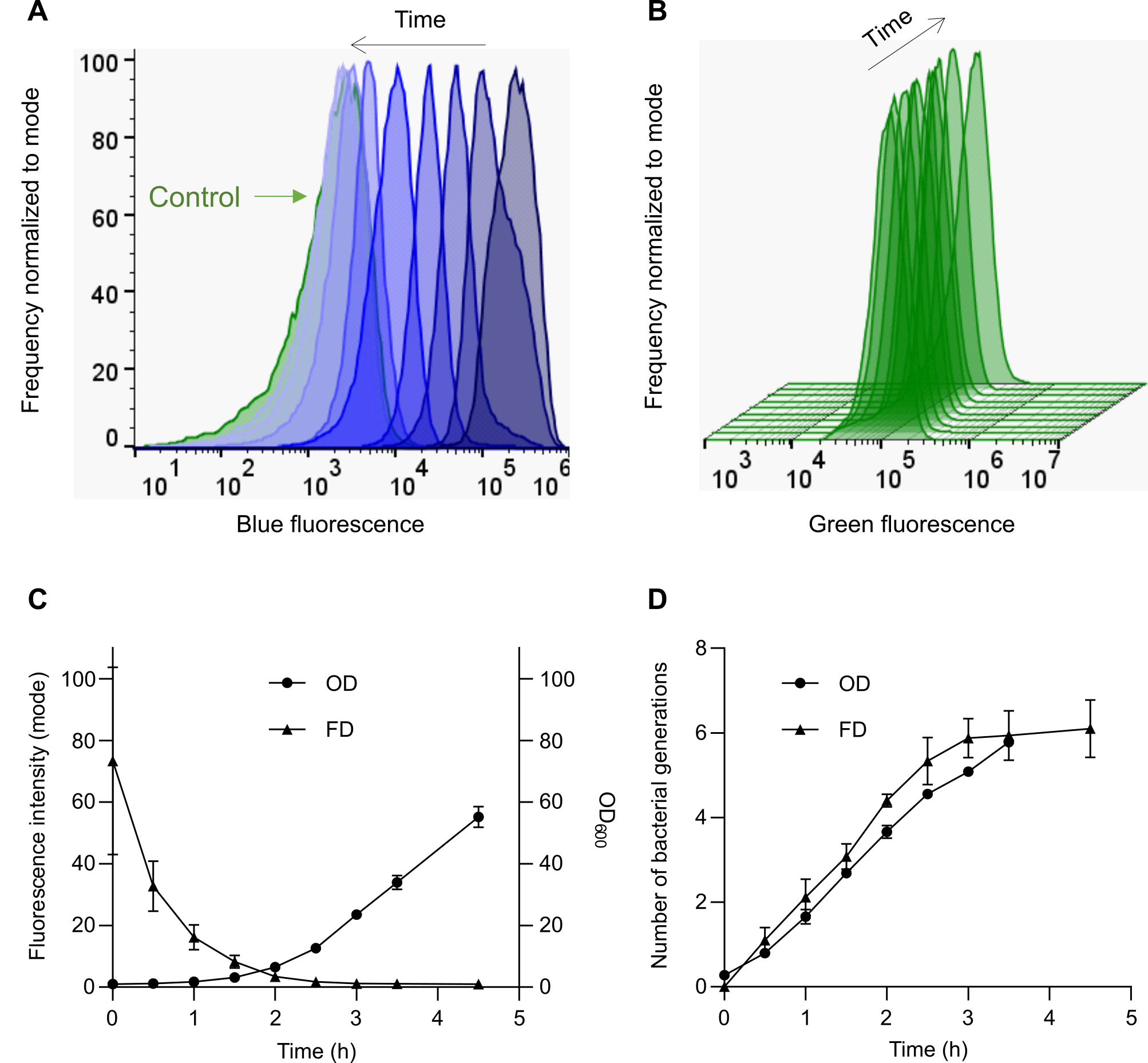

Supplement: S1 Fig — (A-D) S. aureus SH1000 expressing GFP in stationary phase was labeled with eFluor-450 at 10 μM and then diluted in LB medium at 37°C to an initial OD600nm = 0.05. The culture was incubated, and green (GFP) and blue (eFluor-450) fluorescence intensities were analyzed by flow cytometry every 30 minutes, except for the last time point, which was taken at a one-hour interval. OD600nm measurements were taken simultaneously with a spectrophotometer. (A, B) Flow cytometry profile representing the frequency of events normalized to mode as a function of blue (eFluor-450) (A) or green (GFP) (B) intensity over time. It showed either a uniform stepwise halving of blue (eFluor-450) intensity at each time point, indicating a uniform and consistent replication pattern until reaching background noise (A) or a maintained green (GFP) intensity, indicating the stability of GFP expression (B). N > 15500 events per time-point were recorded. (C) Quantifications from flow cytometry and OD600nm measurements normalized to the last and initial time points, respectively. (D) Bacterial replication curves derived from DFD and OD600nm methods normalized by the equation FD(tn) = OD(tn+1) showing similar patterns for 6 generations and doubling times (e.g., 27.6 min for DFD and 31.2 min for OD, calculated between 1 h and 2 h). (C, D) Results were presented as mean ± SD from 3 independent experiments. (TIFF) [file ppat.1013525.s001.tiff]

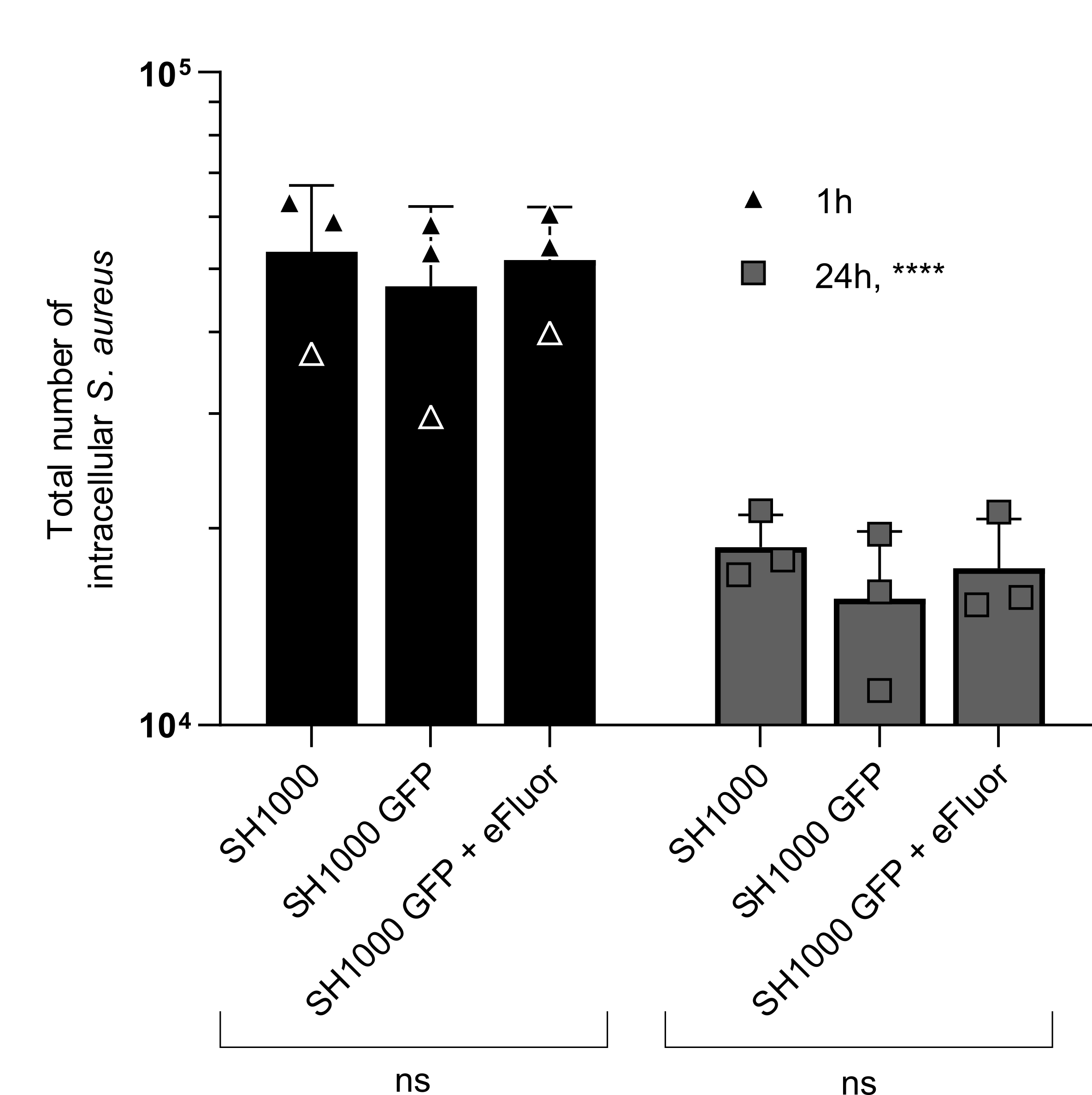

Supplement: S2 Fig — MG63 cells, seeded at sparse density and unlabeled, were infected at an MOI of 8 with either S. aureus SH1000 or SH1000-GFP strains, pre-labeled or unlabeled with eFluor-450. Following 2 hours of co-incubation, lysostaphin at 10 µg/mL was added to eliminate extracellular S. aureus. Intracellular S. aureus were collected at 1 and 24 hours post-infection (hpi), and total number of S. aureus forming colonies on agar plate was assessed showing no significant differences between conditions at each time-points. Results were presented as mean ± SD from 3 independent experiments in technical triplicate. Two-way analysis of variance (ANOVA) test with Sidak’s correction for multiple comparisons post hoc test (ns: treatment, p < 0.0001: time). (TIFF) [file ppat.1013525.s002.tiff]

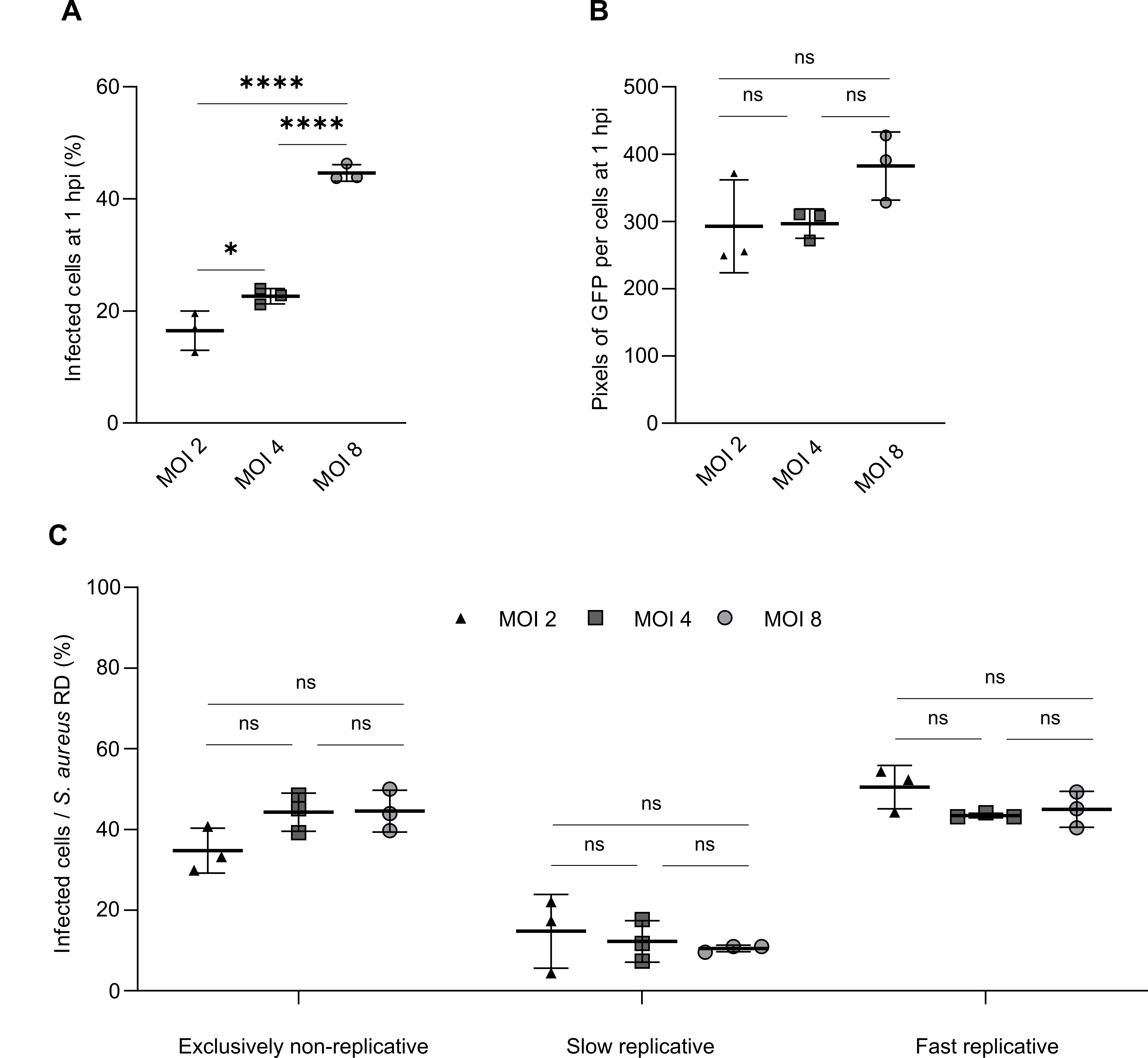

Supplement: S3 Fig — (A-C) MG63 cells, seeded at sparse density and labeled with CellTracker Red CMTPX were infected with S. aureus SH1000 expressing GFP strain pre-labeled by eFluor-450 at an MOI of 2, 4, and 8. Following 2 hours of co-incubation, lysostaphin at 10 µg/mL was added to eliminate extracellular S. aureus. Time-lapse imaging was conducted over 24 hours with hourly acquisitions using automated confocal microscopy. (A) Quantification of the percentage of infected cells. (B) Quantification of the S. aureus load per cell, measured as GFP pixel count per cell. (C) Quantification of infected cells based on the intracellular S. aureus replication dynamics (RD) of SH1000 over the 24-hour infection period. (A-C) Results were presented as mean ± SD from 3 independent experiments in technical triplicate. One-way ANOVA test with Tukey’s correction for multiple comparisons post hoc test: *p < 0.05, ****p < 0.0001. (TIFF) [file ppat.1013525.s003.tiff]

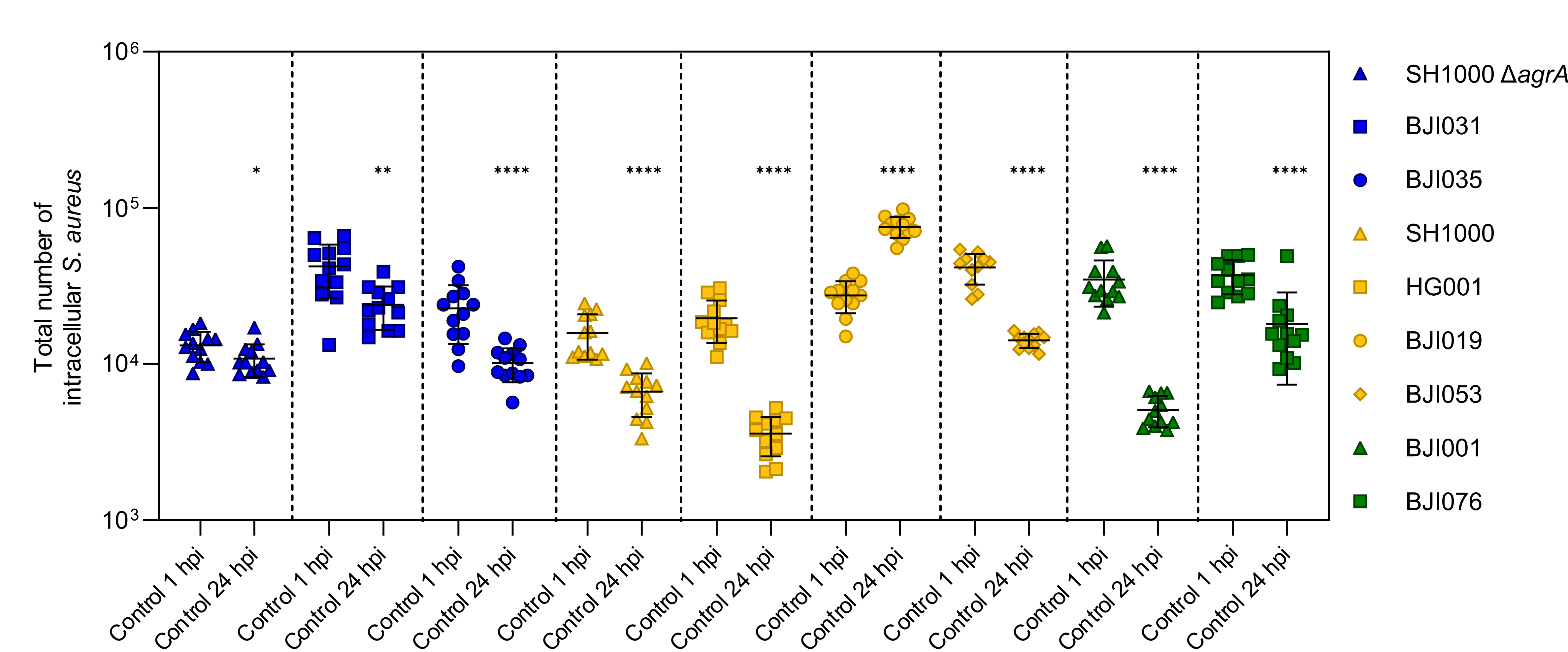

Supplement: S4 Fig — MG63 cells, seeded at confluent density and unlabeled, were infected with a range of S. aureus strains and clinical isolates expressing GFP at MOI 8 pre-labeled by eFluor-450 (S1 Table). Following 2 hours of co-incubation, lysostaphin at 10 µg/mL was added to eliminate extracellular S. aureus. Intracellular S. aureus were collected at 1 hpi and 24 hpi, and the total number of S. aureus forming colonies on agar plate was investigated. Total number of intracellular S. aureus SH1000 forming colonies on plates. Results were presented as mean ± SD representing 12 individual values from 4 independent experiments. Mann-Whitney test: *p < 0.05, **p < 0.01, ****p < 0.0001. (TIFF) [file ppat.1013525.s004.tiff]

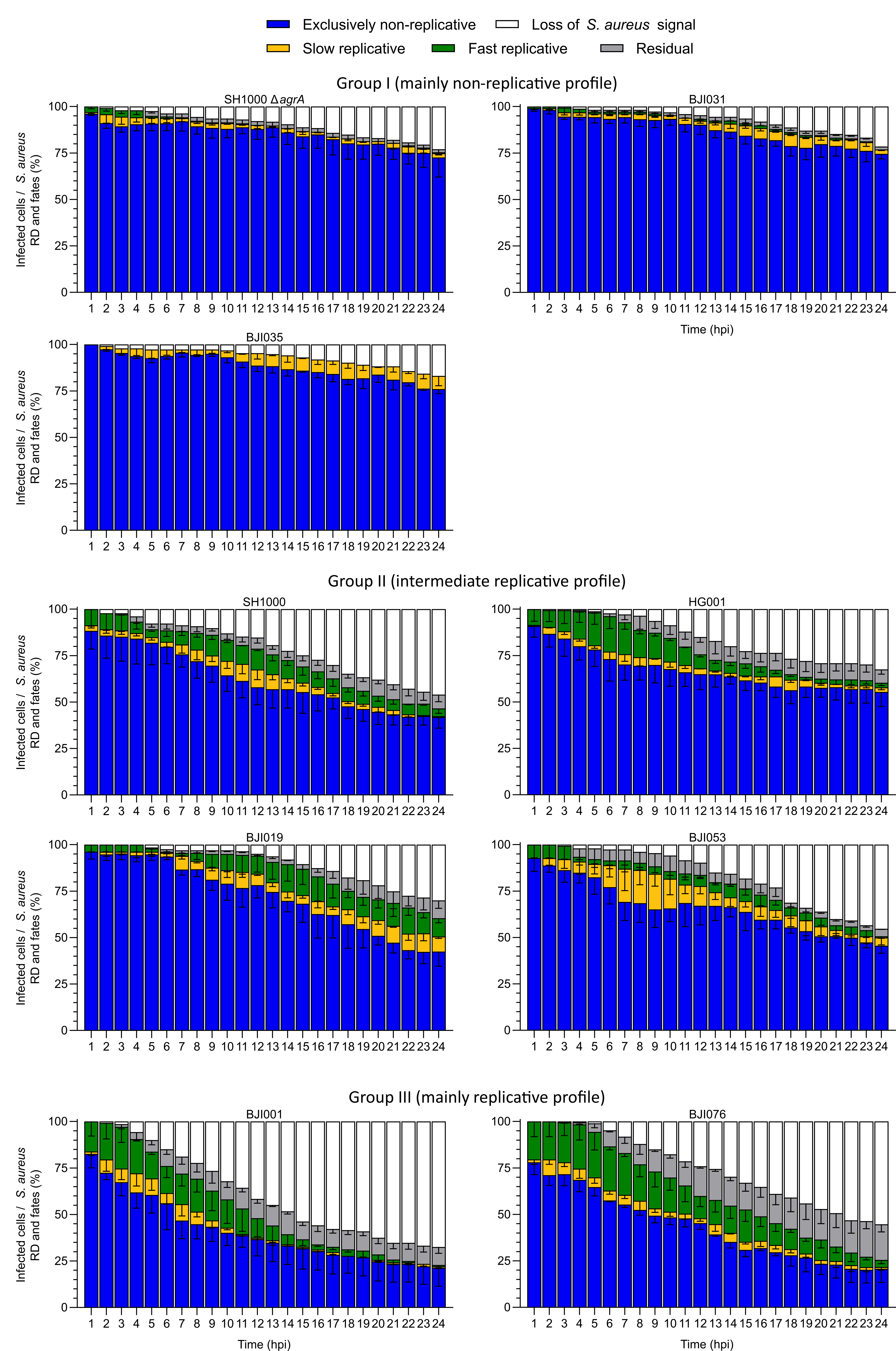

Supplement: S5 Fig — MG63 cells, seeded at sparse density and labeled with CellTracker Red CMTPX, were infected with a range of S. aureus strains and clinical isolates expressing GFP at MOI 8 pre-labeled by eFluor-450 (S1 Table). Following 2 hours of co-incubation, lysostaphin at 10 µg/mL was added to eliminate extracellular S. aureus. Concomitantly, rifampicin was added or not at 6 µg/mL. Time-lapse imaging was conducted over 24 hours with hourly acquisitions using automated confocal microscopy. Quantification of infected cells based on intracellular S. aureus replication dynamics (RD) performed hourly across the 24-hour infection period. Results were presented as mean ± SD from 3 independent experiments in technical triplicate. (TIFF) [file ppat.1013525.s005.tiff]

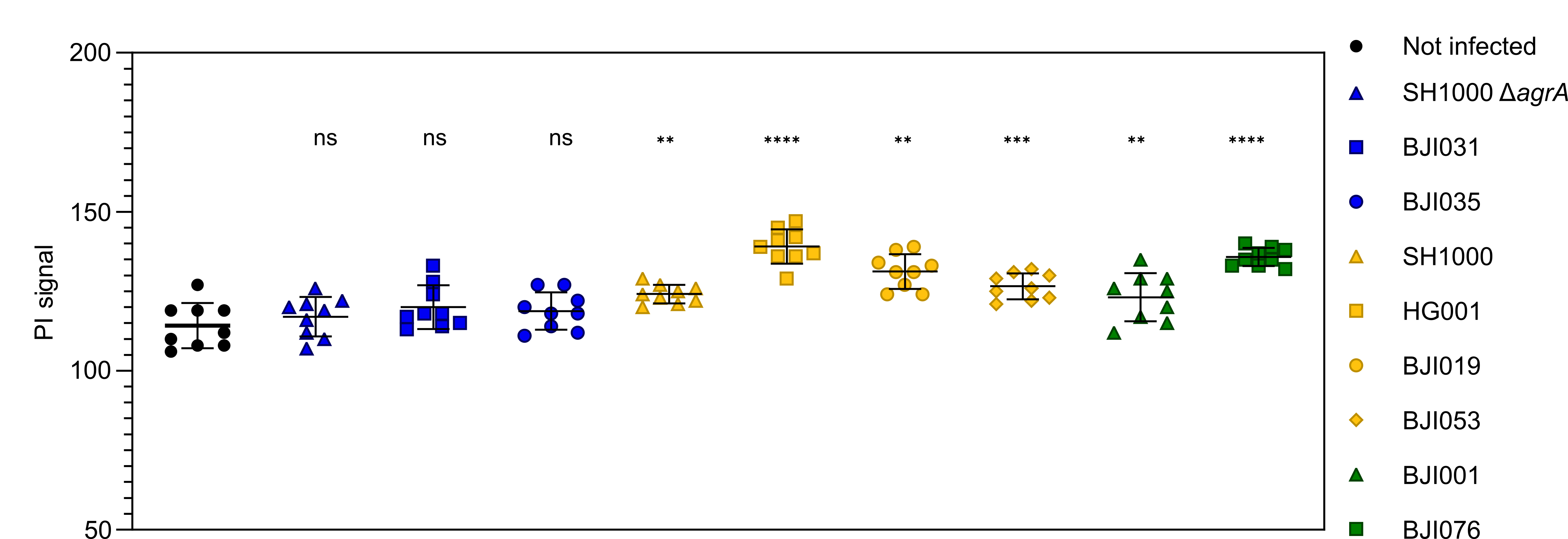

Supplement: S6 Fig — MG63 cells, seeded at confluent density and unlabeled, were infected with a range of S. aureus strains and clinical isolates expressing GFP at MOI 8 (S1 Table). Following 2 hours of co-incubation, lysostaphin at 10 µg/mL was added, to eliminate extracellular S. aureus. Concomitantly, propidium iodide (PI) was added at 2 µg/mL. At 24 hpi PI fluorescence intensity was measured with a plate reader. PI signal indicates S. aureus-induced cytotoxicity trough host cells membrane damage. Results were presented as mean ± SD representing 9 individual values from 3 independent experiments. One-way ANOVA with Dunnett’s correction for multiple post hoc comparisons with the control and Mann-Whitney test: **p < 0.01, ****p < 0.0001. (TIFF) [file ppat.1013525.s006.tiff]

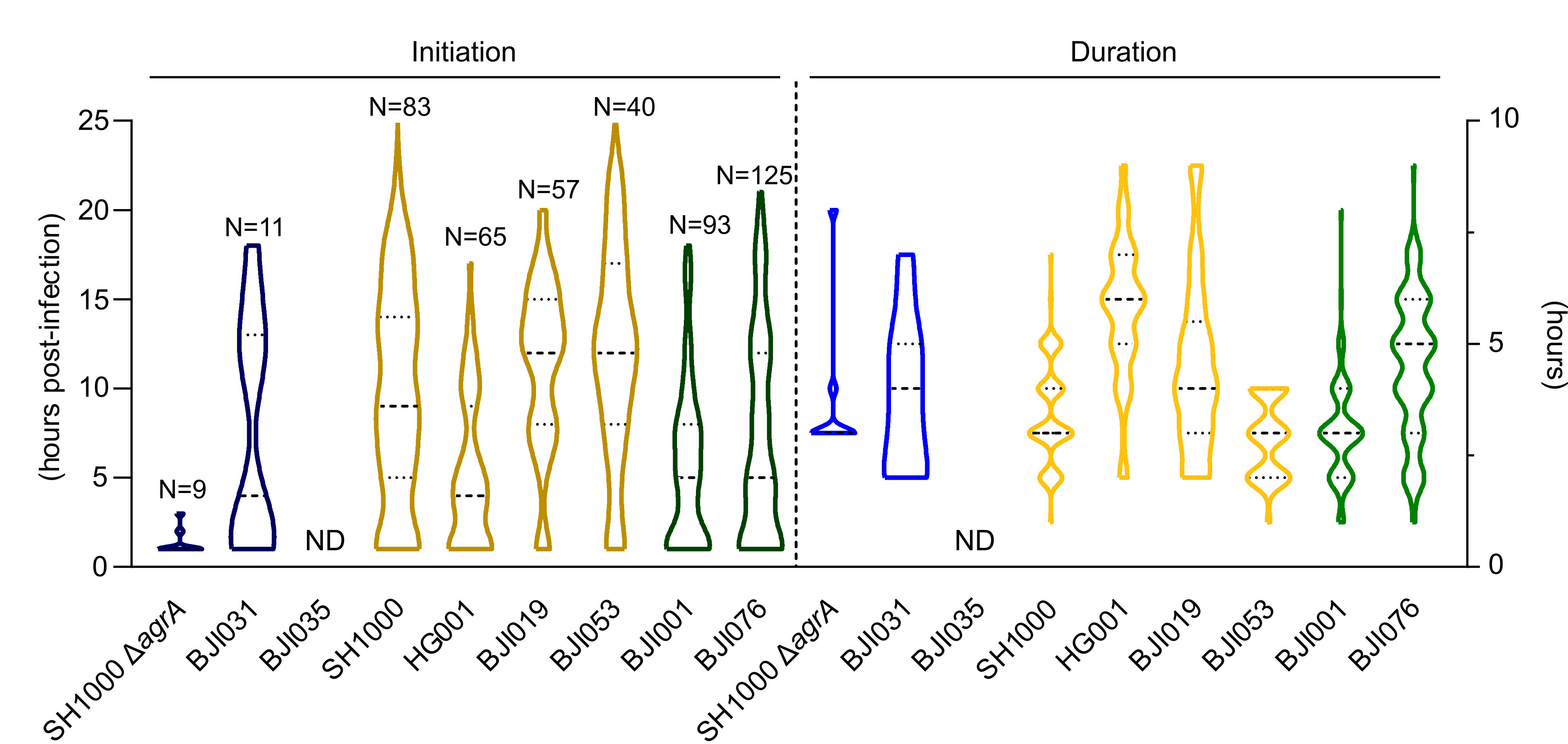

Supplement: S7 Fig — MG63 cells, seeded at sparse density and labeled with CellTracker Red CMTPX, were infected with a range of S. aureus strains and clinical isolates expressing GFP at MOI 8 pre-labeled by eFluor-450 (S1 Table). Following 2 hours of co-incubation, lysostaphin at 10 µg/mL was added to eliminate extracellular S. aureus. Concomitantly, rifampicin was added or not at 6 µg/mL. Time-lapse imaging was conducted over 24 hours with hourly acquisitions using automated confocal microscopy. Quantification of the initiation and duration of the fast replicative phase for each strain, with ongoing fast replicative phases at the end of the observation period excluded from analysis. Results were presented as median and quartiles from 3 independent experiments in technical triplicate. (ND = Not detected). (TIFF) [file ppat.1013525.s007.tiff]

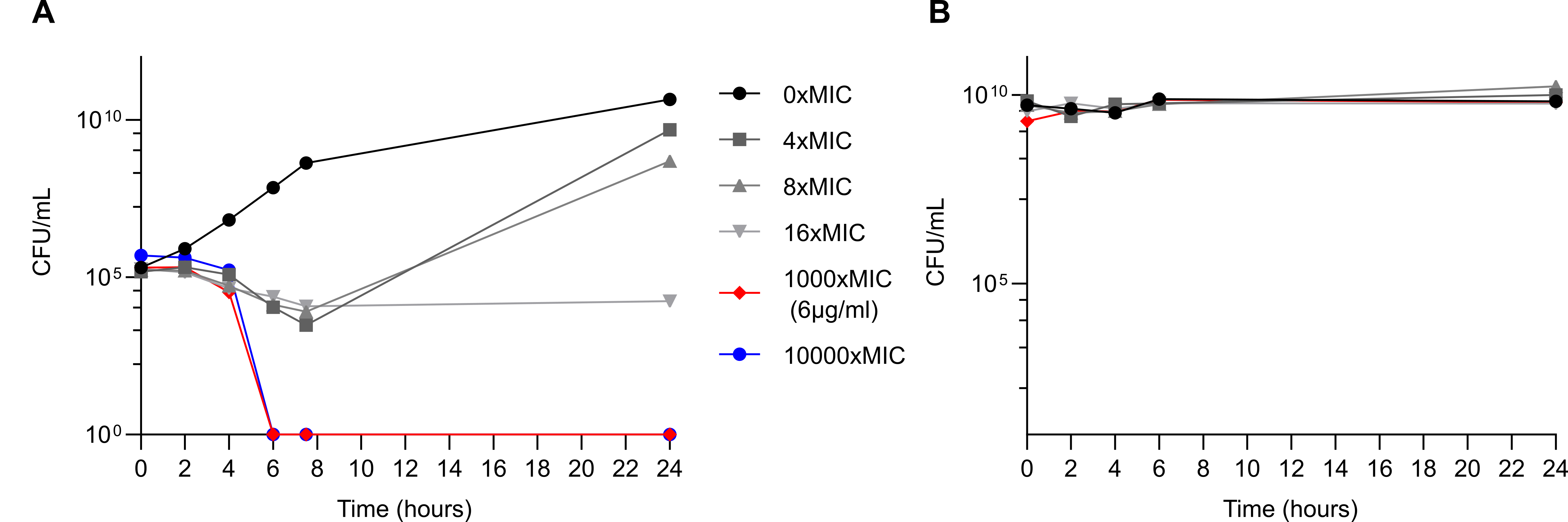

Supplement: S8 Fig — S. aureus SH1000 expressing GFP was cultivated in LB medium to reach either the exponential phase (A) or stationary phase (B). Cultures were subsequently treated with a range of concentrations of rifampicin (0; 4; 8; 16; 1000; and 10,000 times the MIC). Samples were collected at designated intervals, diluted and plated on agar plates to quantify CFU. Total number of S. aureus forming colonies was investigated displaying differences in bacterial survival across concentrations and growth phases. Results represent a single experiment. (TIFF) [file ppat.1013525.s008.tiff]

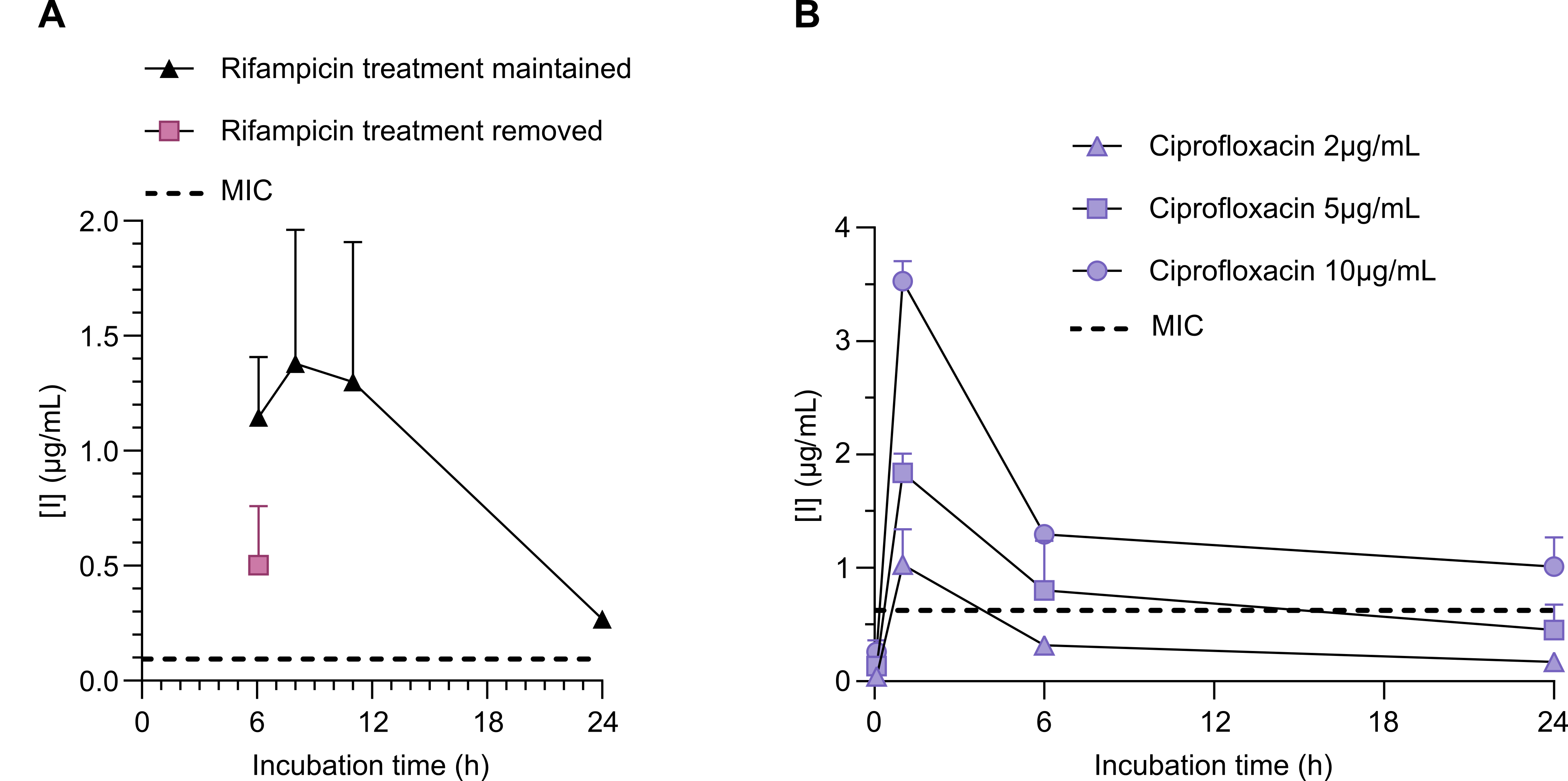

Supplement: S9 Fig — (A, B) MG63 cells, seeded at confluent density and unlabeled, were treated either with rifampicin at 6 µg/mL or ciprofloxacin at 2, 5, and 10 µg/mL. For rifampicin, cells were either washed or left unwashed 6 hours post-treatment, followed by collection after 5 minutes, 2 hours, 5 hours, and 18 hours. For ciprofloxacin, cells were collected at 5 minutes, 1 hour, 6 hours, and 24 hours post-treatment. Intracellular concentrations of rifampicin (A) and ciprofloxacin (B) were measured using mass spectrometry. Results were presented as mean ± SD from 2 independent experiments. (TIFF) [file ppat.1013525.s009.tiff]

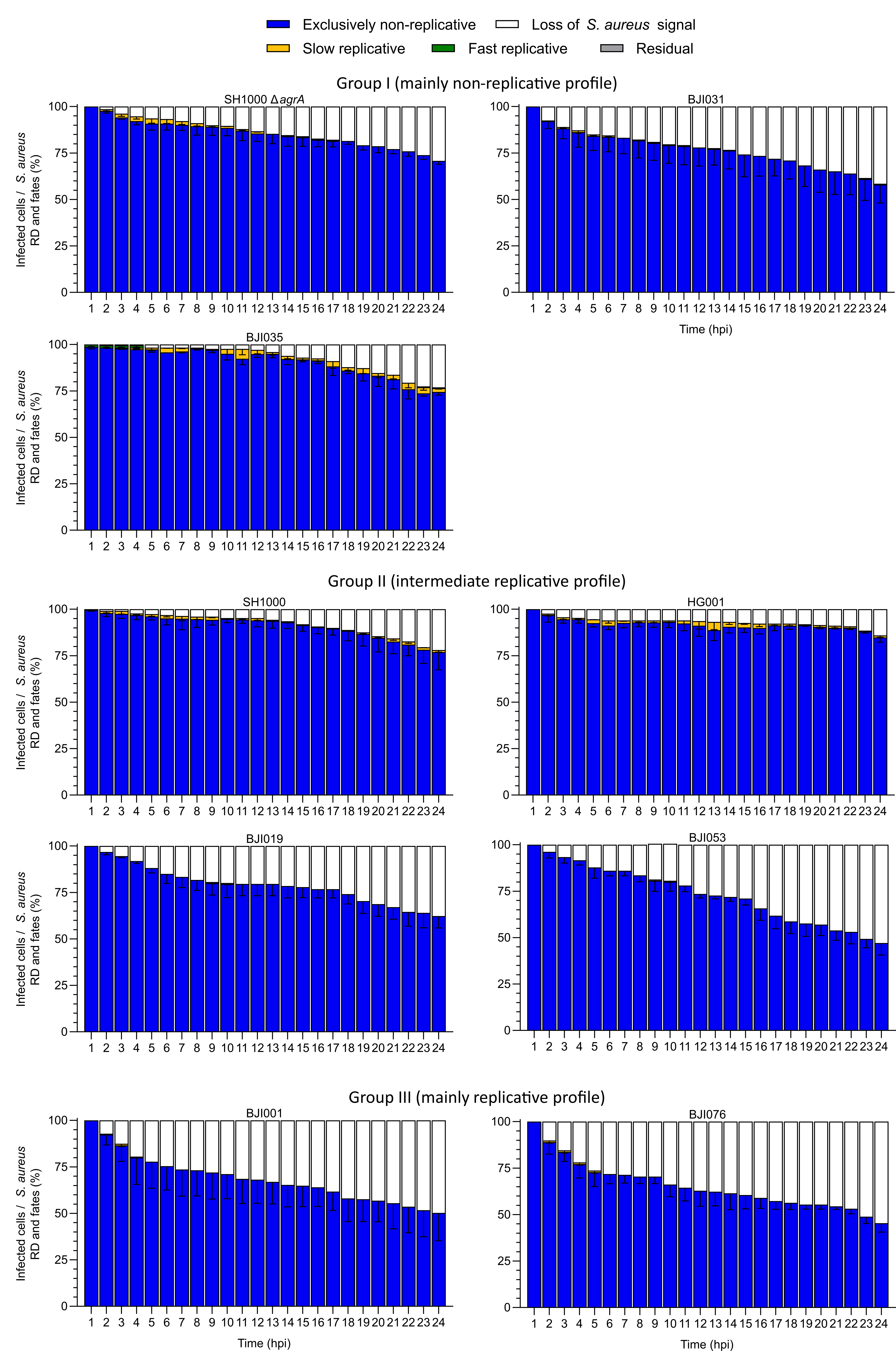

Supplement: S10 Fig — MG63 cells, seeded at sparse density and labeled with CellTracker Red CMTPX, were infected with a range of S. aureus strains and clinical isolates expressing GFP at MOI 8 pre-labeled by eFluor-450 (S1 Table). Following 2 hours of co-incubation, lysostaphin at 10 µg/mL was added to eliminate extracellular S. aureus. Concomitantly, rifampicin was added or not at 6 µg/mL. Time-lapse imaging was conducted over 24 hours with hourly acquisitions using automated confocal microscopy. Quantification of infected cells based on intracellular S. aureus replication dynamics (RD) performed hourly across the 24-hour infection period. Results were presented as mean ± SD from 3 independent experiments in technical triplicate. (TIFF) [file ppat.1013525.s010.tiff]

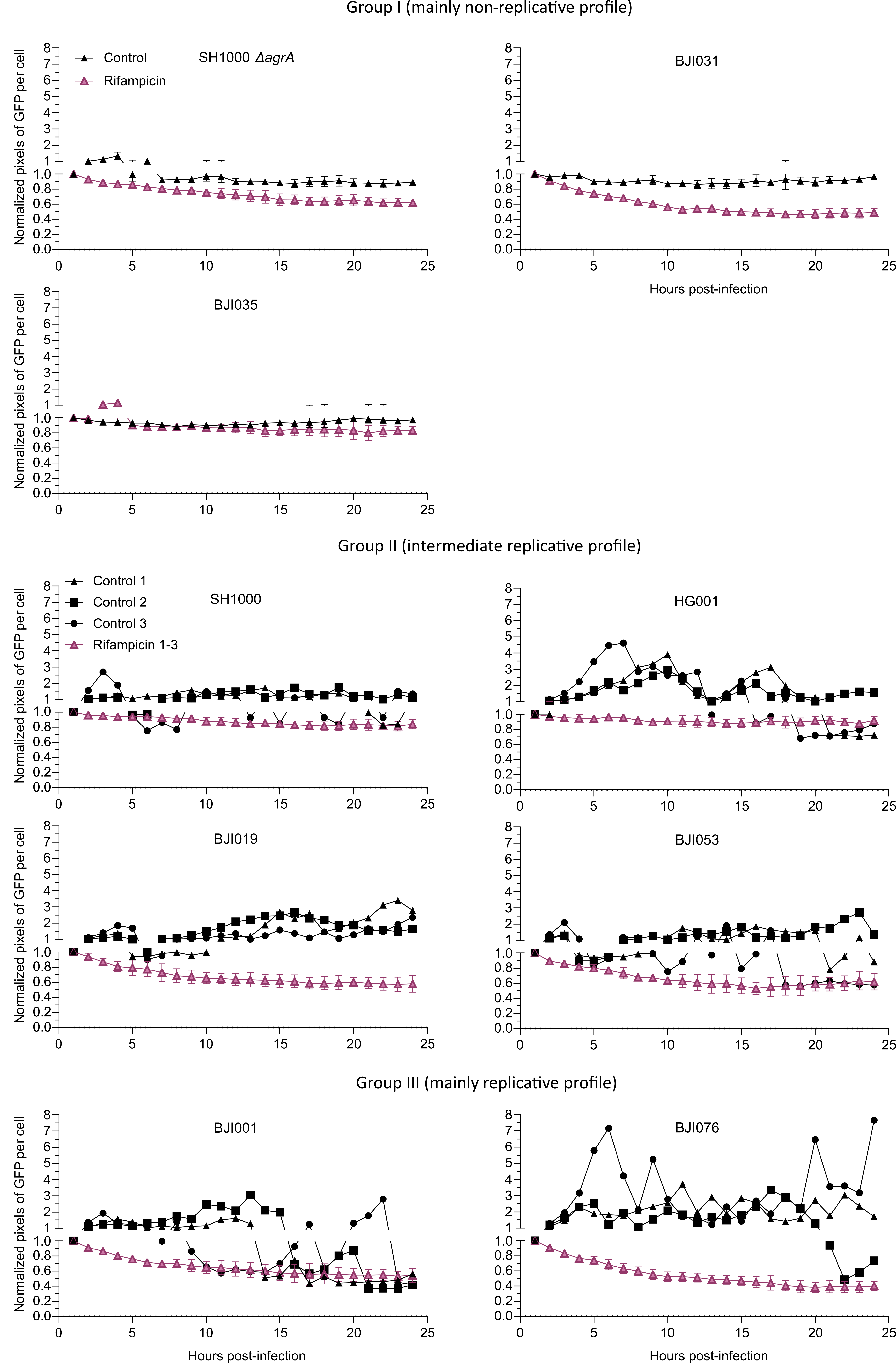

Supplement: S11 Fig — MG63 cells, seeded at sparse density and labeled with CellTracker Red CMTPX, were infected with a range of S. aureus strains and clinical isolates expressing GFP at MOI 8 pre-labeled by eFluor-450 (S1 Table). Following 2 hours of co-incubation, lysostaphin at 10 µg/mL was added to eliminate extracellular S. aureus. Concomitantly, rifampicin was added or not at 6 µg/mL. Time-lapse imaging was conducted over 24 hours with hourly acquisitions using automated confocal microscopy. Quantification of the S. aureus population size per cell over time represented by the total green (GFP) pixel count normalized per cell. Results were presented as individuals or mean ± SD from 3 independent experiments in technical triplicate. (TIFF) [file ppat.1013525.s011.tiff]

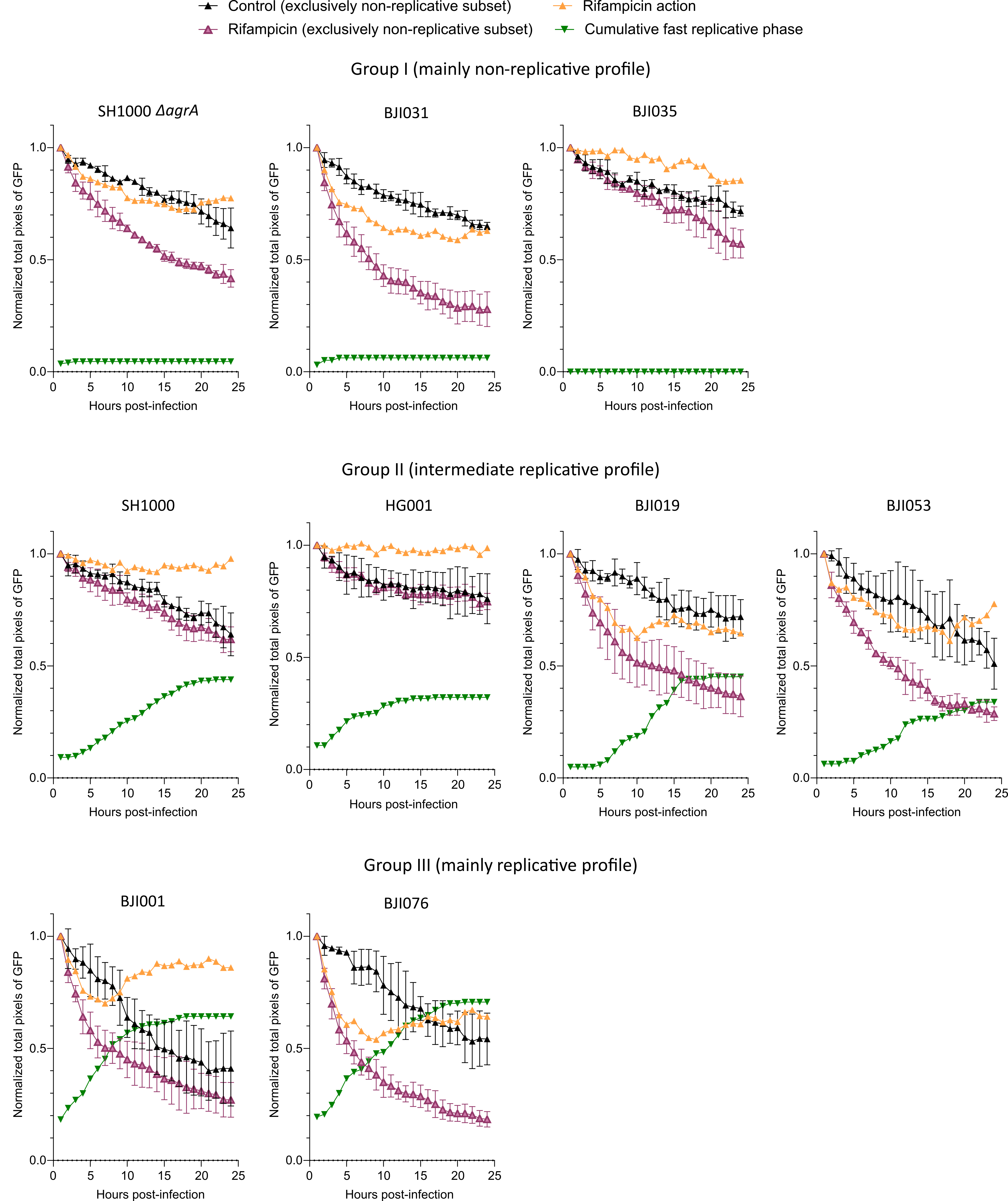

Supplement: S12 Fig — MG63 cells, seeded at sparse density and labeled with CellTracker Red CMTPX, were infected with a range of S. aureus strains and clinical isolates expressing GFP at MOI 8 pre-labeled by eFluor-450 (S1 Table). Following 2 hours of co-incubation, lysostaphin at 10 µg/mL was added to eliminate extracellular S. aureus. Concomitantly, rifampicin was added or not at 6 µg/mL. Time-lapse imaging was conducted over 24 hours with hourly acquisitions using automated confocal microscopy. Quantification of the S. aureus population size, from the exclusively non-replicative osteoblasts subset, over time represented by the total green (GFP) pixel count in the control (black curves) and the rifampicin-treated condition (blue curves). Rifampicin action was calculated through the difference between the control and rifampicin treated conditions (orange curves). Cumulative quantification of infected cells in the control condition experiencing a fast replicative phase (green curves). Results were presented as mean ± SD from 3 independent experiments in technical triplicate. (TIFF) [file ppat.1013525.s012.tiff]

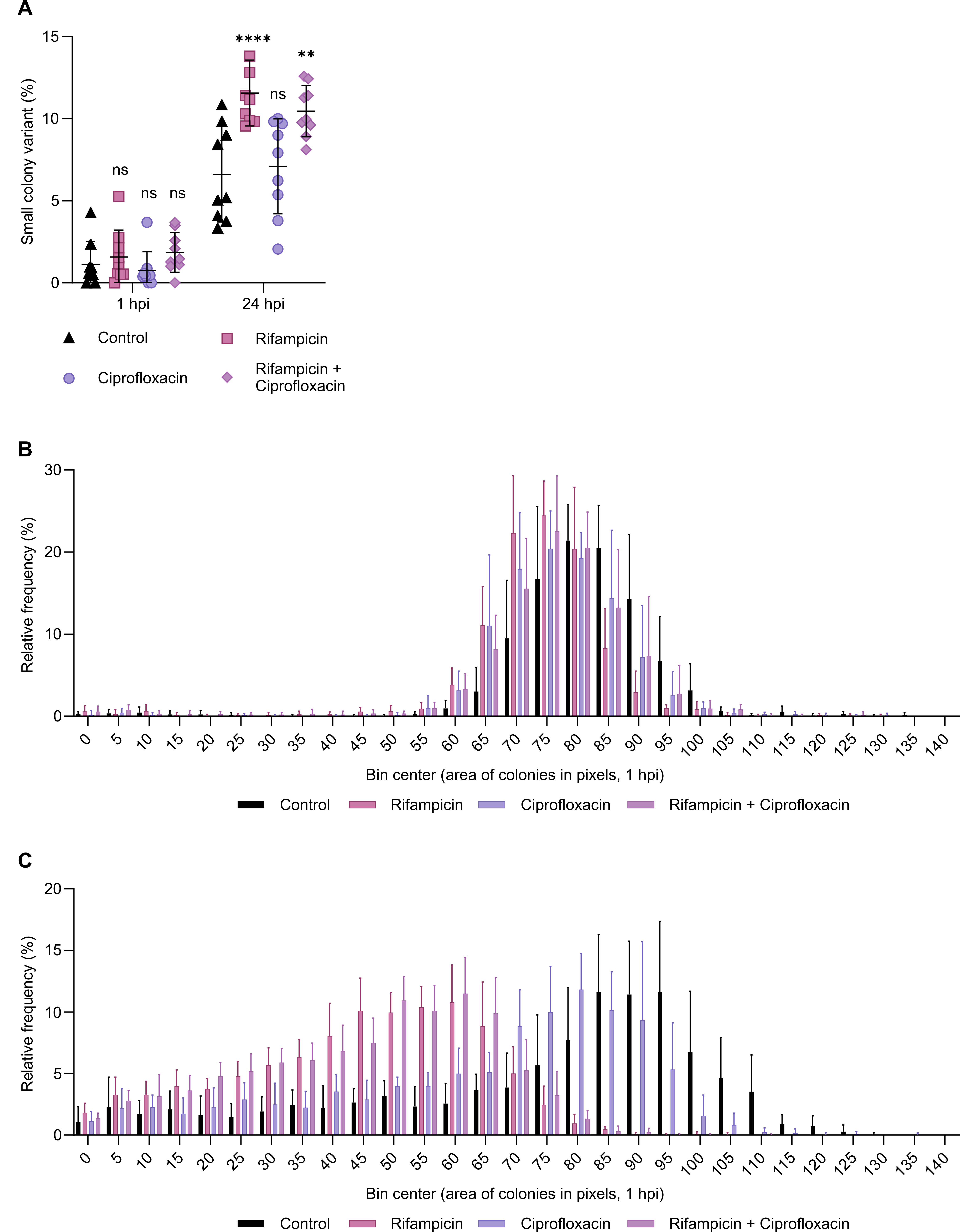

Supplement: S13 Fig — MG63 cells, seeded at sparse density and labeled with CellTracker Red CMTPX, were infected with a range of S. aureus strains and clinical isolates expressing GFP at MOI 8 pre-labeled by eFluor-450 (S1 Table). Following 2 hours of co-incubation, lysostaphin at 10 µg/mL was added to eliminate extracellular S. aureus. Concomitantly, rifampicin and/or ciprofloxacin were added or not at 6 µg/mL and 2 µg/mL, respectively. Intracellular S. aureus were collected at 1 hpi and 24 hpi, and the total number of S. aureus forming colonies on agar plate was investigated. (A) Quantification of the small colony variant (SCV) phenotype, identified as colonies with an area less than 1/5th of the median area of control condition. (B, C) Distribution frequency of colony area of collected intracellular S. aureus at 1 hpi (B) or 24 hpi (C). (A-C) Results were presented as mean ± SD representing 9 individual values (A) from 3 independent experiments (A-C). Two-way ANOVA test with Sidak’s correction for multiple comparisons post hoc test (p < 0.0001: treatment, p < 0.0001: time): **p < 0.01, ****p < 0.0001. (TIFF) [file ppat.1013525.s013.tiff]

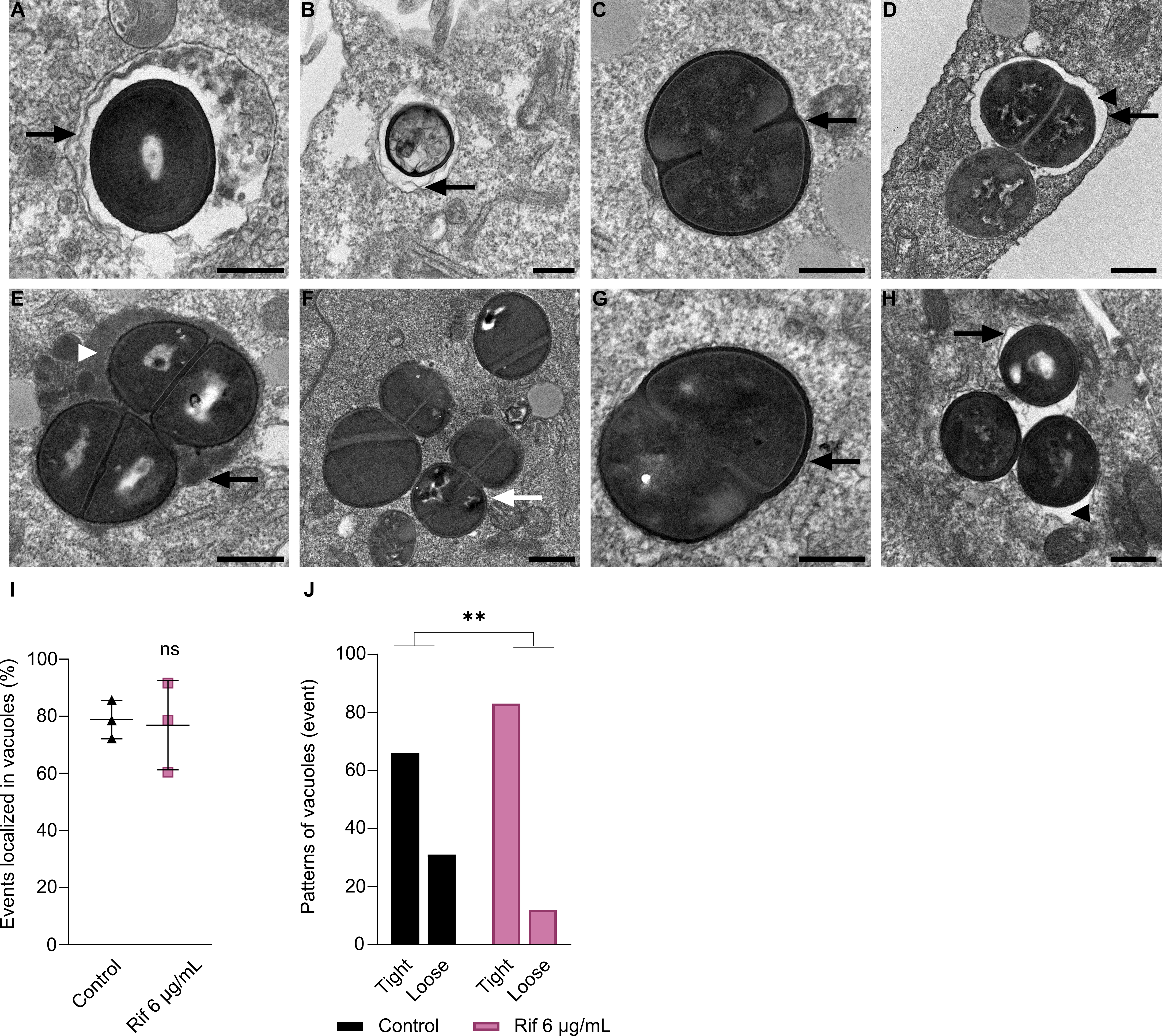

Supplement: S14 Fig — (A-H) Unlabeled MG63 cells were seeded at sparse density and infected at MOI 8 with S. aureus SH1000 expressing GFP pre-labeled by eFluor-450. Following 2 hours of co-incubation, lysostaphin at 10 µg/mL was added to eliminate extracellular S. aureus. Concomitantly, cells were left untreated (A-F) or treated with rifampicin at 6 µg/mL (G, H). At 24 hpi cells were fixed and imaged by transmission electron microscopy (TEM). (A-H) Representative TEM images showing intracellular S. aureus residing either within a multilamellar (A, B; black arrow) or a single-membrane vacuole (C-E, G, H; black arrow) or in the cytoplasm (F; white arrow). The intra-vacuolar space is either clear (D, H; black arrowhead) or dense (E; white arrowhead) to the electrons (scale bar = 0.4 µm) I, J Corresponding microscopy quantification of events where isolated or small cluster of 2–3 S. aureus localized within vacuoles (I) and events where the vacuoles are tightly associated with the bacteria (J). Results were presented as mean ± SD representing 3 individual values from 3 independent experiments. Control = 124 events, 179 bacteria; rifampicin = 126 events, 172 bacteria. Mann-Whitney test (I) or chi-square test (J): **p < 0.01. (TIFF) [file ppat.1013525.s014.tiff]

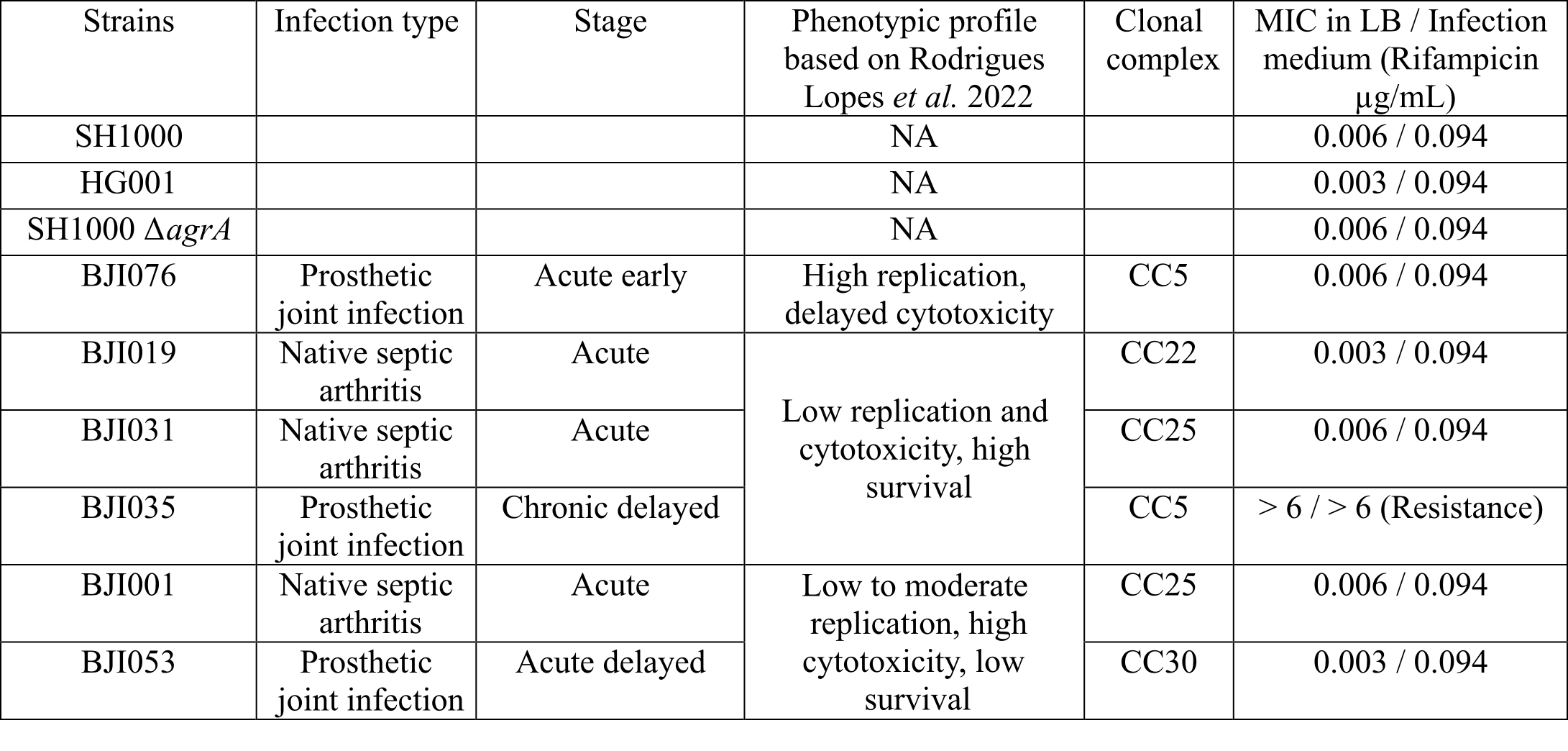

Supplement: S1 Table — (TIFF) [file ppat.1013525.s015.tiff]

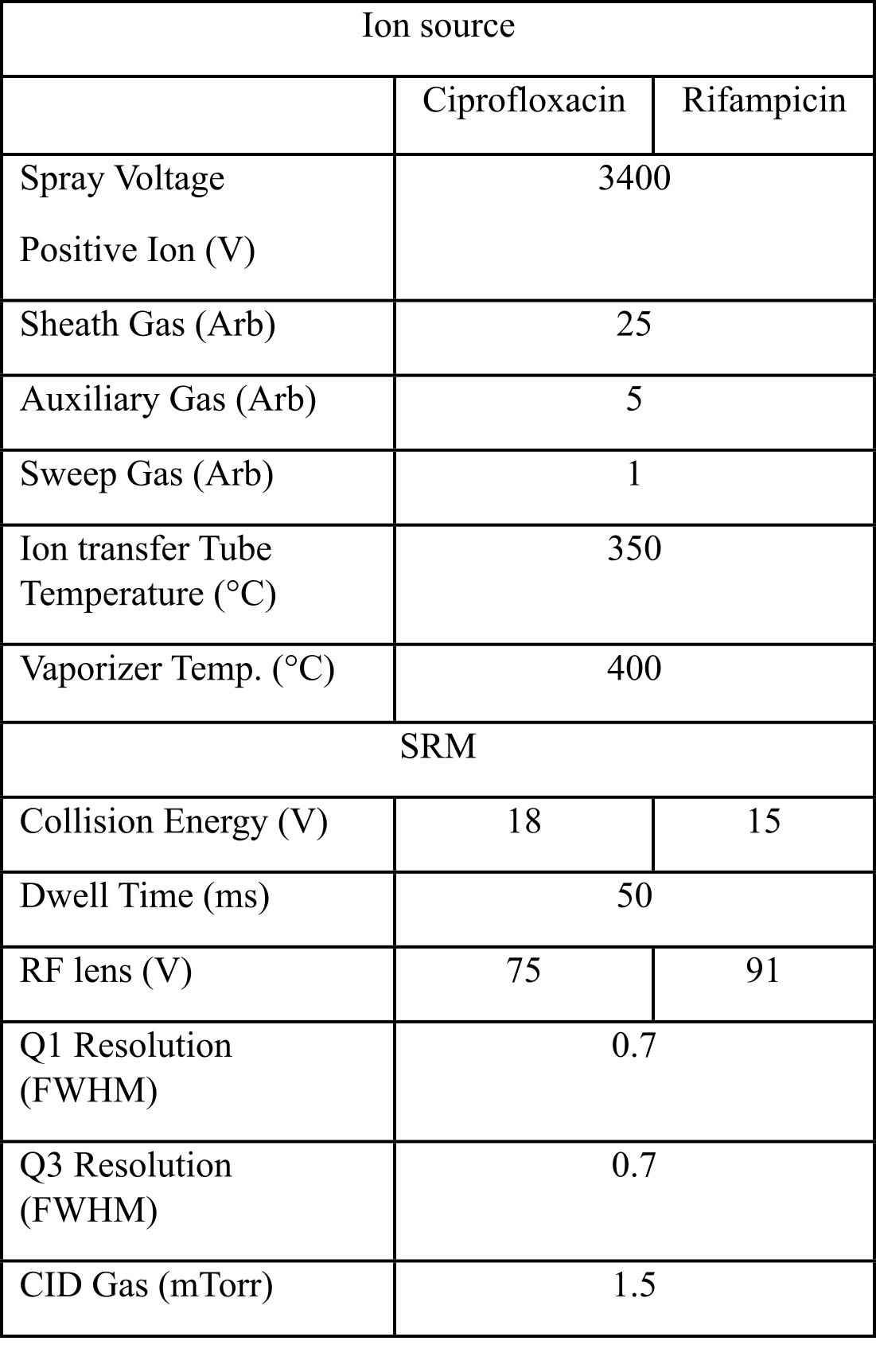

Supplement: S2 Table — (TIFF) [file ppat.1013525.s016.tiff]
